# Effect of Asymptomatic and Symptomatic COVID-19 on Acute Ischemic Stroke Revascularization Outcomes

**DOI:** 10.1161/STROKEAHA.123.043899

**Published:** 2023-12-12

**Authors:** Davide Strambo, João Pedro Marto, George Ntaios, Thanh N. Nguyen, Patrik Michel

**Affiliations:** Stroke Center, Neurology Service, Department of Neurological Sciences, Lausanne University Hospital, University of Lausanne, Switzerland (D.S., P.M.).; Department of Neurology, Hospital de Egas Moniz, Centro Hospitalar Lisboa Ocidental, Portugal (J.P.M.).; Departement of Internal Medicine, Faculty of Medicine, University of Thessaly, Larissa, Greece (G.N.).; Departement of Neurology, Boston Medical Center, MA (T.N.N.).; Neurology, Comprehensive Stroke Center, Charles University Faculty of Medicine and University Hospital; 2nd Department of Neurology, Institute of Psychiatry and Neurology; Neurology, Leuven University Hospital; Alexandria University Hospitals and Affiliated Stroke Network; School of Biomedical Engineering and Imaging Sciences, St Thomas Hospital, King’s College London; Neurology, Neurosurgery, UPMC; Stroke Center, Neurology Service, Department of Neurological Sciences, Lausanne University Hospital and University of Lausanne; Neurology, University Hospital of Zürich; Neurology, University Hospital of Zürich; Neurology Clinic, Stroke Center, Neurocenter of Southern Switzerland, Ente Ospedaliero Cantonale; Neurology Clinic, Stroke Center, Neurocenter of Southern Switzerland, Ente Ospedaliero Cantonale; Stroke Center, Neurology, Inselspital, Bern University Hospital, University of Bern; Stroke Center, Neurology, Inselspital, Bern University Hospital, University of Bern; Stroke Center, Geneva University Hospital; Neuroradiology, Geneva University Hospital; Stroke Center, University Hospital Basel, University of Basel; Stroke Center, University Hospital Basel, University of Basel; Stroke Center, University Hospital Basel, University of Basel; Stroke Center, Kantonsspital Lucerne; Stroke Center, Hirslanden Hospital; Stroke Center, Hirslanden Hospital; Stroke Center, Neurology Service, Department of Neurological Sciences, Lausanne University Hospital and University of Lausanne; Neuroradiology, Hospital de Egas Moniz, Centro Hospitalar Lisboa Ocidental; Neurology, Centro Hospitalar Universitário de Coimbra; Neurology, Centro Hospitalar Universitário de Coimbra; Neuroradiology, Centro Hospitalar Universitário de Coimbra; Stroke Unit, Hospital de São José, Centro Hospitalar Universitário Lisboa Central; Stroke Unit, Neurology, Hospital de Santa Maria, Centro Hospitalar Universitário Lisboa Norte; Stroke Unit, Neurology, Hospital de Santa Maria, Centro Hospitalar Universitário Lisboa Norte; Neuroradiology, Hospital de Santa Maria, Centro Hospitalar Universitário Lisboa Norte; Neuroradiology, Hospital de Santa Maria, Centro Hospitalar Universitário Lisboa Norte; Neuroradiology, Hospital de Santa Maria, Centro Hospitalar Universitário Lisboa Norte; Neuroradiology, Hospital de Santa Maria, Centro Hospitalar Universitário Lisboa Norte; Neurology, Centro Hospitalar Universitário São João; Neurology, Centro Hospitalar Universitário São João; Neuroradiology, Centro Hospitalar Universitário São João; Neuroradiology, Centro Hospitalar Universitário São João; Neurology, Hospital de Braga; Neuroradiology, Hospital de Braga; Neurology, Hospital Garcia de Orta; Neurology, Hospital Garcia de Orta; Neuroradiology, Centro Hospitalar de Vila Nova de Gaia/Espinho; Neuroradiology, Centro Hospitalar de Vila Nova de Gaia/Espinho; Neurology, Centro Hospitalar de Vila Nova de Gaia/Espinho; Neurology, Unidade Local de Saúde de Matosinhos; Neurology, Unidade Local de Saúde de Matosinhos; Neurology, Centro Hospitalar Universitário do Porto; Stroke Unit, Azienda Ospedaliera Universitaria Integrata; Stroke Unit, Azienda Ospedaliera Universitaria Integrata; Stroke Unit, Azienda Ospedaliera Universitaria Integrata; IRCCS Istituto delle Scienze Neurologiche di Bologna, Neurology and Stroke Center, Maggiore Hospital; IRCCS Istituto delle Scienze Neurologiche di Bologna, Neurology and Stroke Center, Maggiore Hospital; IRCCS Istituto delle Scienze Neurologiche di Bologna, Neurology and Stroke Center, Maggiore Hospital; IRCCS Istituto delle Scienze Neurologiche di Bologna, Neurology and Stroke Center, Maggiore Hospital; Neurology, ASST Papa Giovanni XXIII; Department of Medicine and Surgery, University of Parma, Italy; Stroke Care Program, Department of Emergency, Parma University Hospital, Italy; Clinical and Experimental Sciences, Neurology Clinic, University of Brescia; Neurology and Stroke Unit, Azienda Socio Sanitaria Territoriale; Neurology Unit, Stroke Unit, Azienda Unità Sanitaria-IRCCS di Reggio Emilia; Neuroradiology Unit, Azienda Unità Sanitaria-IRCCS di Reggio Emilia; Neurology, San Gerardo Hospital, Department of Medicine and Surgery and Milan Center for Neuroscience, University of Milano Bicocca; Neurology, San Gerardo Hospital, Department of Medicine and Surgery and Milan Center for Neuroscience, University of Milano Bicocca; Stroke Unit, Fondazione IRCCS Ca’ Granda Ospedale Maggiore Policlinico; Neurology, Policlinico Universitario Agostino Gemelli; Emergency Neurology and Stroke Unit, IRCCS Humanitas Clinical and Research Center; Neurology, Hôpital Fondation Adolphe De Rothschild; Neurology, Hôpital Fondation Adolphe De Rothschild; Interventional Neuroradiology, Hôpital Fondation Adolphe De Rothschild; Interventional Neuroradiology, Hôpital Fondation Adolphe De Rothschild; Interventional Neuroradiology, Hôpital Fondation Adolphe De Rothschild; Interventional Neuroradiology, Center Hospitalier Régional Universitaire, Hôpital Jean Minjoz; Interventional Neuroradiology, Center Hospitalier Régional Universitaire, Hôpital Jean Minjoz; University Grenoble Alpes, Stroke Unit; University Grenoble Alpes, Stroke Unit; University Grenoble Alpes, Stroke Unit; University of Bordeaux, Bordeaux University Hospital, Stroke Unit; University of Bordeaux, Bordeaux University Hospital, Stroke Unit; University of Bordeaux, Bordeaux University Hospital, Stroke Unit; Diagnostic and Interventional Neuroradiology, University Medical Center-Hamburg-Eppendorf; Diagnostic and Interventional Neuroradiology, University Medical Center-Hamburg-Eppendorf; Diagnostic and Interventional Neuroradiology, University Medical Center-Hamburg-Eppendorf; Neurology, University Hospital Frankfurt, Goethe University; Neurology, University Hospital Frankfurt, Goethe University; Neurology, Center for Stroke Research, Berlin Institute of Health, Charité-Universitätsmedizin Berlin; Neurology, Center for Stroke Research, Berlin Institute of Health, Charité-Universitätsmedizin Berlin; Neuroradiology, Charité-Universitätsmedizin Berlin; Neurology, St. John’s Hospital; Neurology, St. John’s Hospital; Neurology, St. John’s Hospital; Neurology, Medical University of Innsbruck; Neurology, Medical University of Innsbruck; Neuroradiology, Medical University of Innsbruck; Neurology, Amsterdam University Medical Centers, Amsterdam Neuroscience; Neurology, Amsterdam University Medical Centers, Amsterdam Neuroscience; Neurology, Amsterdam University Medical Centers, Amsterdam Neuroscience; Neurology, Amsterdam University Medical Centers, Amsterdam Neuroscience; Neurology, Haaglanden Medical Center, Hague; Radiology, Leiden University Medical Center; Neurology, Haaglanden Medical Center, Hague; Radiology, Leiden University Medical Center; Neurology, Leuven University Hospital; Neurology, Universitair Ziekenhuis Brussel, Center for Neurosciences, Vrije Universiteit Brussel; Neurology, Universitair Ziekenhuis Brussel, Center for Neurosciences, Vrije Universiteit Brussel; Neurology, Stroke Unit, Europe Hospitals; Neurology, Stroke Unit, Europe Hospitals; Neurology, Centre Hospitalier Universitaire de Charleroi; Neurology, Centre Hospitalier Universitaire de Charleroi; Neurology and Stroke Center, Hospital Universitario 12 de Octubre, Instituto de Investigación Hospital 12 de Octubre; Neurology and Stroke Center, Hospital Universitario 12 de Octubre, Instituto de Investigación Hospital 12 de Octubre; Neurology and Stroke Center, Hospital Universitario 12 de Octubre, Instituto de Investigación Hospital 12 de Octubre; Neurology and Stroke Center, Hospital Universitario 12 de Octubre, Instituto de Investigación Hospital 12 de Octubre; Neurology, Ramón y Cajal University Hospital; Neurology, Ramón y Cajal University Hospital; Neurology, Ramón y Cajal University Hospital; Neurology, Ramón y Cajal University Hospital; Neurology and Stroke Center, La Paz University Hospital, Institute for Health Research-IdiPAZ; Neurology and Stroke Center, La Paz University Hospital, Institute for Health Research-IdiPAZ; Neurology and Stroke Center, La Paz University Hospital, Institute for Health Research-IdiPAZ; Neurology and Stroke Center, La Paz University Hospital, Institute for Health Research-IdiPAZ; Neurology, Hospital Universitario Virgen Macarena; Neurology, Hospital Universitario Virgen Macarena; Stroke Center, Hospital General Universitario Gregorio Marañón; Stroke Unit, Germans Trias i Pujol Hospital; Stroke Unit, Germans Trias i Pujol Hospital; Stroke Unit, Germans Trias i Pujol Hospital; Neurology, Comprehensive Stroke Center, Hospital Clinic From Barcelona; Neurology, Comprehensive Stroke Center, Hospital Clinic From Barcelona; Neurology, Comprehensive Stroke Center, Hospital Clinic From Barcelona; Neurology, Complejo Hospitalario Universitario de Albacete; Neurology, Complejo Hospitalario Universitario de Albacete; Neurology, Complejo Hospitalario Universitario de Albacete; Stroke Unit, Neurology, and Interventional Neuroradiology Unit, Radiology, Hospital Universitario Miguel Servet; Stroke and Geriatric Medicine, Aintree University Hospital; Comprehensive Stroke Service, University College London Hospitals NHS Foundation Trust and Stroke Research Centre, University College London; Comprehensive Stroke Service, University College London Hospitals NHS Foundation Trust and Stroke Research Centre, University College London; University College London, Queen Square Institute of Neurology; Neurology, Akershus University Hospital, Lørenskog and Department of General Practice, University of Oslo; Clinical Neuroscience, Institute of Neuroscience and Physiology, Sahlgrenska Academy at University of Gothenburg; Neurology, Sahlgrenska University Hospital, Region Västra Götaland; Clinical Neuroscience, Institute of Neuroscience and Physiology, Sahlgrenska Academy at University of Gothenburg; Neurology, Sahlgrenska University Hospital, Region Västra Götaland; Radiology, Institute of Clinical Sciences, Sahlgrenska Academy at the University of Gothenburg; Interventional and Diagnostic Neuroradiology, Sahlgrenska University Hospital, Region Västra Götaland; Neurology, Comprehensive Stroke Center, Charles University Faculty of Medicine and University Hospital; Neurology, Comprehensive Stroke Center, Charles University Faculty of Medicine and University Hospital; Radiology, Comprehensive Stroke Center, Charles University Faculty of Medicine and University Hospital, Hradec Králové, Czech Republic; Radiology, Comprehensive Stroke Center, Charles University Faculty of Medicine and University Hospital, Hradec Králové, Czech Republic; Radiology, Comprehensive Stroke Center, Charles University Faculty of Medicine and University Hospital, Hradec Králové, Czech Republic; International Clinical Research Center and Department of Neurology, St. Anne’s University Hospital, Faculty of Medicine at Masaryk University; Neurology, University Hospital Ostrava; Neurology, University Hospital Ostrava; Neurology, University Hospital Ostrava; Neurology, České Budějovice Hospital; Neurology, České Budějovice Hospital; Neurology, Jihlava Hospital; Neurology, Jihlava Hospital; Neurocenter, Regional Hospital Liberec; Neurocenter, Regional Hospital Liberec; Neurocenter, Regional Hospital Liberec; Cerebrovascular Center, Na Homolce Hospital; Cerebrovascular Center, Na Homolce Hospital; Cerebrovascular Center, Na Homolce Hospital; Neurology, Karviná Miners Hospital, Inc; Cerebrovascular Center, University Hospital in Motol; Cerebrovascular Center, University Hospital in Motol; Cerebrovascular Center, University Hospital in Motol; Cerebrovascular Center, Central Military Hospital; Cerebrovascular Center, Central Military Hospital; Cerebrovascular Center, Central Military Hospital; Cerebrovascular Center, Central Military Hospital; Cerebrovascular Center, General University Hospital; Cerebrovascular Center, General University Hospital; 2nd Department of Neurology, Institute of Psychiatry and Neurology; 2nd Department of Neurology, Institute of Psychiatry and Neurology; 1st Department of Neurology, Institute of Psychiatry and Neurology; 1st Department of Neurology, Institute of Psychiatry and Neurology; Neurology, University Hospital, Jagiellonian University; Neurology, University Hospital, Jagiellonian University; Neurology, University Hospital, Jagiellonian University; Neurology, University Hospital, Jagiellonian University; Neurology, University Hospital, Jagiellonian University; Neurology, Institute of Medical Sciences, Medical College of Rzeszów University; Neurology, Institute of Medical Sciences, Medical College of Rzeszów University; Neurology, Institute of Medical Sciences, Medical College of Rzeszów University; Neurology, Institute of Medical Sciences, Medical College of Rzeszów University; Neurology and Stroke, St. John Paul II Western Hospital, Grodzisk Mazowiecki; Neurology and Stroke, St. John Paul II Western Hospital, Grodzisk Mazowiecki; Neurology, National Medical Institute of the Ministry of Interior and Administration; Neurology, National Medical Institute of the Ministry of Interior and Administration; Neurology, Wroclaw Medical University; Neurology, Wroclaw Medical University; Neurology, Wroclaw Medical University; Neurology, Wroclaw Medical University; Radiology, Wroclaw Medical University; Neurosurgery and Neurology, Nicolaus Copernicus University in Torun Ludwik Rydygier Collegium Medicum; Stroke Intervention Center, Neurosurgery and Neurology, Jan Biziel University Hospital; Stroke Intervention Center, Neurosurgery and Neurology, Jan Biziel University Hospital; Neurology, Institute of Medical Sciences, University of Opole; Clinic of Neurology, Military Institute of Medicine; Clinic of Neurology, Military Institute of Medicine; Clinic of Neurology, Military Institute of Medicine; Neurology, University of Warmia and Mazury; Radiology, Provincial Specialist Hospital; Neurology, University Emergency Hospital Bucharest, University of Medicine and Pharmacy “Carol Davila; Neurology, University Emergency Hospital Bucharest, University of Medicine and Pharmacy “Carol Davila; Neurology, University Emergency Hospital Bucharest, University of Medicine and Pharmacy “Carol Davila,”; Neurology, University Emergency Hospital Bucharest, University of Medicine and Pharmacy “Carol Davila,”; Radiology, University Emergency Hospital Bucharest; Neurology and Stroke Unit, Elias University Emergency Hospital, University of Medicine and Pharmacy “Carol Davila; Neurology and Stroke Unit, Elias University Emergency Hospital, University of Medicine and Pharmacy “Carol Davila,” Bucharest; Neurology and Stroke Unit, Elias University Emergency Hospital, University of Medicine and Pharmacy “Carol Davila,”; Neurology, Eskisehir Osmangazi University; Ain Shams University Affiliated Saudi German Hospital; Ain Shams University Affiliated Saudi German Hospital; Neuropsychiatry Department, Tanta University; Neurology, Ibn Sina Hospital; Neurology, Ibn Sina Hospital; Neurology, Jaber Al-Ahmad Hospital; Neurology, School of Medicine, Zanjan University of Medical Sciences; Stroke Unit, Neurology, Hillel Yaffe Medical Center; Neurology, Radiology, Boston Medical Center, MA; Neurology, Radiology, Boston Medical Center, MA; Neurology, Radiology, Boston Medical Center, MA; Neurosurgery, Thomas Jefferson University Hospital, Philadelphia, PA; Neurosurgery, Thomas Jefferson University Hospital, Philadelphia, PA; Neurosurgery, Thomas Jefferson University Hospital, Philadelphia, PA; Neurosurgery, Thomas Jefferson University Hospital, Philadelphia, PA; Neurology, Neurosurgery, UPMC; Neurology, Henry Ford Hospital; Neurology, Henry Ford Hospital; Neurosciences, Spectrum Health and Michigan State University, East Lansing; Neurosciences, Spectrum Health and Michigan State University, East Lansing; Neurosciences, Spectrum Health and Michigan State University, East Lansing; Neurosciences, Spectrum Health and Michigan State University, East Lansing; Neurology, University of Arkansas for Medical Sciences, Little Rock; Neurology, University of Arkansas for Medical Sciences, Little Rock; Neurology, Upstate University Hospital, Syracuse, NY; Neurology, Upstate University Hospital, Syracuse, NY; Neurology, Upstate University Hospital, Syracuse, NY; Neurology, University of Kansas Medical Center, Lawrence, KS; Neurology, University of Kansas Medical Center, Lawrence, KS; Endovascular Neurological Surgery and Neurology, Rutgers, The State University of New Jersey, Newark; Endovascular Neurological Surgery and Neurology, Rutgers, The State University of New Jersey, Newark; Endovascular Neurological Surgery and Neurology, Rutgers, The State University of New Jersey, Newark; Endovascular Neurological Surgery and Neurology, Rutgers, The State University of New Jersey, Newark; Neurology, Wayne State University, Detroit Medical Center, MI; Neurology, Wayne State University, Detroit Medical Center, MI; Neurology, Wayne State University, Detroit Medical Center, MI; Stroke Clinic, Instituto Nacional de Neurologia y Neurocirugia Manuel Velasco Suarez; Stroke Clinic, Instituto Nacional de Neurologia y Neurocirugia Manuel Velasco Suarez; Neurology, Fundación Valle del Lili; Centro de Investigaciones Clínicas, Fundación Valle del Lili; Centro de Investigaciones Clínicas, Fundación Valle del Lili; Neurology, Hospital Nacional Edgardo Rebagliati Martins; Hospital General San Juan de Dios; Neurology, Hospital Nossa Senhora da Conceição Hospital; Neurology, Hospital Nossa Senhora da Conceição Hospital; Ramos Mejía Hospital, Stroke Unit, Buenos Aires; St. Luke’s Medical Center; Neurology, Grant Medical College and Sir JJ Hospital; Neurology, Grant Medical College and Sir JJ Hospital; Neurology, National Health Insurance Service Ilsan Hospital Korea

**Keywords:** endothelial cells, ischemic stroke, renin-angiotensin system, SARS-CoV-2, thrombosis

## Abstract

**BACKGROUND:**

The association of COVID-19 with higher bleeding risk and worse outcomes in acute ischemic stroke (AIS) undergoing revascularization may be related to the presence of infection symptoms. We aimed to assess the safety and outcomes of revascularization treatments in patients with AIS with asymptomatic COVID-19 (AS-COVID) or symptomatic COVID-19 (S-COVID).

**METHODS:**

We conducted an international multicenter retrospective cohort study of consecutive AIS tested for SARS-CoV-2, receiving intravenous thrombolysis and endovascular treatment between 2020 and 2021. We compared COVID-negative controls, AS-COVID, and S-COVID using multivariable regression. We assessed symptomatic intracranial hemorrhage (symptomatic intracerebral hemorrhage), mortality, and 3-month disability (modified Rankin Scale score).

**RESULTS:**

Among 15 124 patients from 105 centers (median age, 71 years; 49% men; 39% treated with intravenous thrombolysis only; and 61% with endovascular treatment±intravenous thrombolysis), 849 (5.6%) had COVID-19, of whom 395 (46%) were asymptomatic and 454 (54%) symptomatic. Compared with controls, both patients with AS-COVID and S-COVID had higher symptomatic intracerebral hemorrhage rates (COVID-controls, 5%; AS-COVID, 7.6%; S-COVID, 9.4%; adjusted odds ratio [aOR], 1.43 [95% CI, 1.03–1.99]; aOR, 1.63 [95% CI, 1.14–2.32], respectively). Only in patients with symptomatic infections, we observed a significant increase in mortality at 24 hours (COVID-controls, 1.3%; S-COVID, 4.8%; aOR, 2.97 [95% CI, 1.76–5.03]) and 3 months (COVID-controls, 19.5%; S-COVID, 40%; aOR, 2.64 [95% CI, 2.06–3.37]). Patients with COVID-19 had worse 3-month disability regardless of disease symptoms although disability was affected to a greater extent in symptomatic patients (aOR for worse modified Rankin Scale score shift: AS-COVID, 1.25 [95% CI, 1.03–1.51]; S-COVID, 2.10 [95% CI, 1.75–2.53]). S-COVID had lower successful recanalization (74.9% versus 85.6%; *P*<0.001), first pass recanalization (20.3% versus 28.3%; *P*=0.005), and a higher number of passes.

**CONCLUSIONS:**

In AIS undergoing revascularization treatments, both AS-COVID and S-COVID influence the risk of intracranial bleeding and worse clinical outcomes. The magnitude of this effect is more pronounced in symptomatic infections, which also present less favorable recanalization outcomes. These findings emphasize the impact of SARS-CoV-2 infection on the prognosis of revascularized AIS independent of symptom status.

**REGISTRATION:**

URL: https://www.clinicaltrials.gov; Unique identifier: NCT04895462.

SARS-CoV-2 infection is associated with a high incidence of thrombotic and cerebrovascular complications.^1–3^

**See related article, p 89**

The pathogenesis of this prothrombotic state is mediated by several mechanisms, including direct viral invasion of endothelial cells, immune-mediated thrombosis and hypercoagulopathy, activation of the alternative renin-angiotensin system pathway, and, for cerebrovascular complications, viral-mediated damage to the neurovascular unit.^4–6^

Previous studies showed that patients with acute ischemic stroke (AIS) and COVID-19 have a worse functional outcome than those without SARS-CoV-2 infection.^7–12^ Similarly, our previous analysis of the Global COVID-19 Stroke Registry showed that patients with AIS and COVID-19 receiving intravenous thrombolysis (IVT) and endovascular treatment (EVT) had higher rates of cerebral bleeding and worse short-term and 3-month outcomes compared with contemporary AIS controls without COVID-19.^13^

Currently, it is unknown whether the higher rates of bleeding complications and worse outcomes after AIS with revascularization treatment are related to the presence of symptoms of COVID-19 infection.

Here, we aimed to assess the safety, clinical, and technical outcomes of revascularization treatment in patients with AIS and asymptomatic COVID-19 (AS-COVID) or symptomatic COVID-19 (S-COVID) in a multicenter, international cohort by comparing with a contemporary control group of patients with no COVID-19 with AIS from the same centers.

## METHODS

### Data Availability

The raw, anonymized data of this study are available from the corresponding author upon reasonable request and after signing a data transfer and user agreement.

### Study Design, Patient Selection, and Study Variables

This retrospective, international, cohort study was conducted on the Global COVID-19 Stroke Registry.^13^ The registry included consecutive patients with AIS receiving IVT and EVT up to 24 hours from the last time seen well. Patients were treated according to stroke guidelines or the local practice of each center. Each participating center included at least 1 patient with COVID-19 and AIS treated with IVT and EVT. The inclusion period of the registry was March 1, 2020, to June 30, 2021.

Patients with COVID-19 were defined as (1) patients with community-acquired SARS-CoV-2 infection confirmed by a positive polymerase chain reaction (PCR) or antigen test, independent of the presence of COVID-19–related symptoms; (2) patients hospitalized due to COVID-19 with an in-hospital stroke; and (3) patients with COVID-19–compatible symptoms before revascularization treatment with positive PCR or antigen test within the first 7 days after treatment. The control group of COVID-19-negative patients included patients without COVID-19–compatible symptoms and with a negative PCR or antigen test within the first 7 days after revascularization treatment.

The registry had the following exclusion criteria: (1) patient without a PCR or antigen test within the first 7 days after treatment; (2) patient with nosocomial SARS-CoV-2 infection after receiving revascularization treatment, defined as PCR or antigen tests becoming positive >7 days after treatment^14^; (3) patient with a suspected/probable case of SARS-CoV-2 infection according to the World Health Organization definition^15^; (4) patient with symptomatic SARS-CoV-2 infection with symptom resolution >7 days before revascularization treatment; and (5) patient with asymptomatic SARS-CoV-2 infection with revascularization treatment performed >10 days after the first positive test for SARS-CoV-2.

For this study, among patients who were originally included in the Global COVID-19 Stroke Registry, we further excluded patients with COVID-19 without information on the presence or absence of symptoms related to the infection.

Each center was required to indicate whether patients exhibited symptoms of COVID-19 at the time of stroke onset or within 7 days prior, following the case definitions provided by the World Health Organization (Table S1).^15^

The reporting is in accordance with the Strengthening of the Reporting of Observational Studies in Epidemiology guidelines.^16^

### Standard Protocol Approvals and Registration

Data from each participating center were anonymized and sent to the coordinating center (Lausanne University Hospital). According to the local ethics committee regulations and national laws, each center was responsible for obtaining ethical approval for data collection and international data sharing. Informed consent was waived due to the retrospective nature of this study with anonymized data. This study was conducted according to the principles of the Declaration of Helsinki. In the coordinating center in Lausanne, approval of the institutional review board and patient consent was not required according to the Swiss Federal Act on Research Involving Human Beings (2011, Human Research Act, Article 3) as all data were anonymized and the project involved assessing safety and quality of routine AIS management in participating centers. The study was registered under https://www.clinicaltrials.gov (NCT04895462).

### Outcomes

For the main outcome, we defined symptomatic intracerebral hemorrhage (SICH) according to ECASS-2 ([European Cooperative Acute Stroke Study]; ≥4-point worsening in the NIHSS score attributable to parenchymal hemorrhage).^17^ As secondary outcomes, we defined symptomatic subarachnoid hemorrhage (SSAH; ≥4-point worsening in the NIHSS score attributable to subarachnoid hemorrhage), any symptomatic intracranial hemorrhage (SICH/SSAH; combination of SICH and SSAH), 24-hour mortality, 3-month mortality, 3-month modified Rankin Scale (mRS), favorable 3-month outcome (mRS score ≤2 or equal to prestroke mRS score), the presence of any radiological hemorrhagic transformation, recanalization after EVT measured by modified Treatment in Cerebral Infarction at the last angiography series, successful recanalization after EVT as final modified Treatment in Cerebral Infarction score ≥2b, the number of passes during EVT, and recanalization at first pass.^18^ All outcomes were assessed locally at each center by the investigators, unblinded to the patient’s COVID status during the acute phase of the stroke.

### Statistical Analysis

We summarized continuous variables as median values with interquartile range and categorical variables as absolute numbers and percentages. We compared baseline and outcome variables between AS-COVID, S-COVID, and COVID-19 negative groups using the Pearson χ^2^ test for categorical variables and the Mann-Whitney *U* test for continuous variables, as appropriate. Multiple pairwise comparison between groups was corrected using the Dunnett method. We performed all analyses of outcomes on the overall cohort and the 2 treatment subgroups: IVT only and EVT.

To assess the association of AS-COVID and S-COVID with the primary outcome (SICH) and with 5 of the secondary outcomes (SSAH, SICH/SSAH, 24-hour and 3-month mortality, 3-month mRS score, and favorable 3-month outcome), we performed multivariable regression analysis for each outcome. For the remaining secondary outcomes, we performed a univariable analysis.

**Figure 1.**
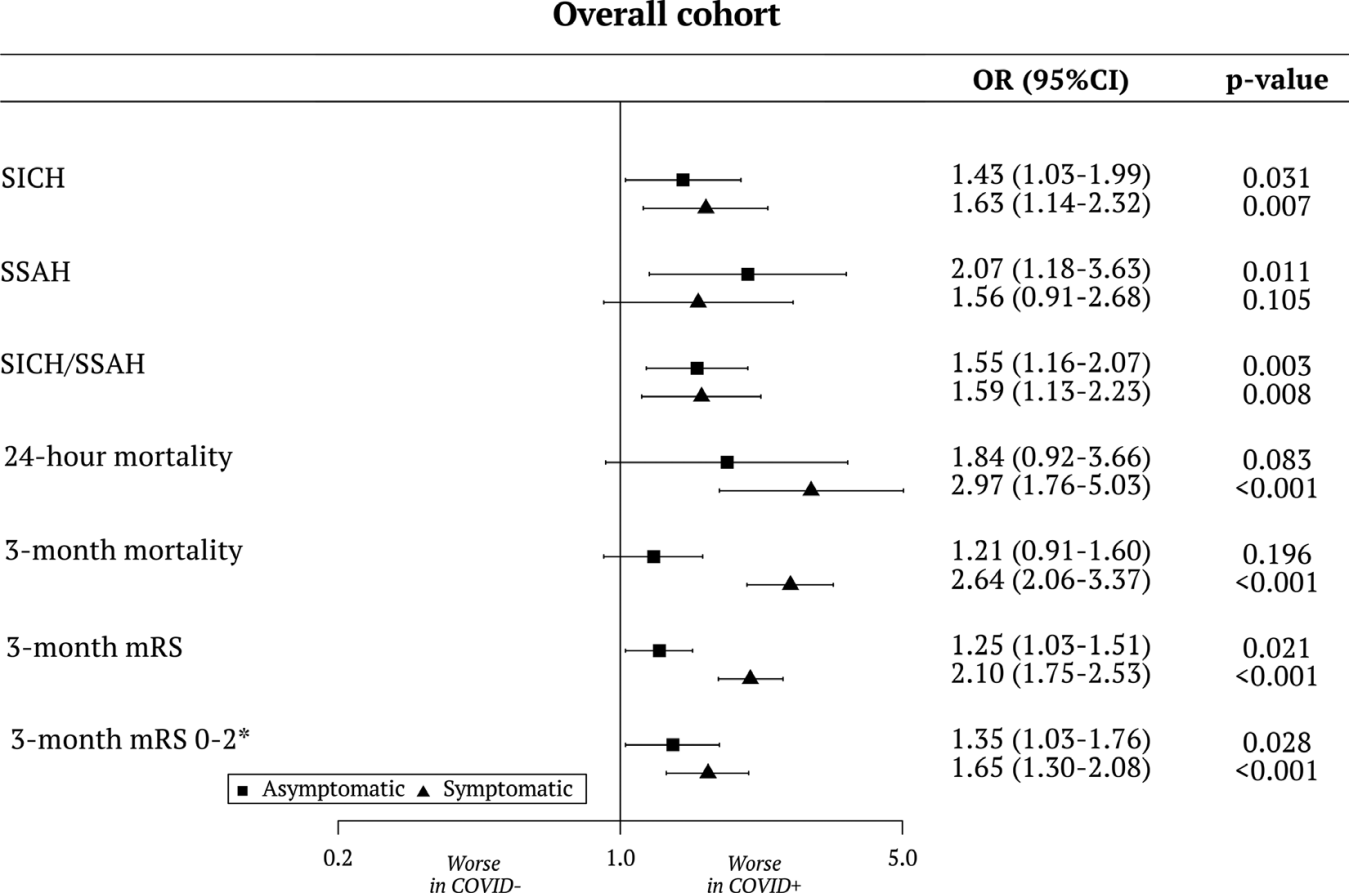
**Forest plot of intracranial bleeding complications, mortality, and disability comparing patients with asymptomatic and symptomatic COVID-19 with COVID-negative controls.** All models were adjusted for age, sex, National Institutes of Health Stroke Scale score, Alberta Stroke Program Early Computed Tomography Score, blood glucose, site of arterial occlusion, tandem lesion, time to treatment (last-time-seen-well-to-needle for intravenous thrombolysis [IVT] and last-time-seen-well-to-groin delay for endovascular treatment [EVT] when applicable), and center volume. Symptomatic intracerebral hemorrhage (SICH), symptomatic subarachnoid hemorrhage (SSAH), and SICH/SSAH models were also adjusted for systolic blood pressure and previous antithrombotic therapy. Mortality and modified Rankin Scale (mRS) score models were adjusted for prestroke mRS score, cancer, and coronary heart disease. Models on the overall cohort were adjusted for revascularization treatment (IVT only vs EVT). Models on the EVT cohort were adjusted for IVT, the number of passes, and successful revascularization. OR indicates odds ratio. *Or return to prestroke mRS, in patients with prestroke mRS>2.

Regarding the multivariable analyses, for the binary outcomes, the model was a logistic regression model, while it was an ordered logit regression model for a 3-month mRS score. The models were adjusted for prespecified potential confounders identified from the previous literature as variables known to be associated with the outcome of interest, namely, age, sex, NIHSS score, Alberta Stroke Program Early Computed Tomography Score, blood glucose, site of arterial occlusion, tandem lesion, time to treatment (last-time-seen-well-to-needle for IVT and last-time-seen-well-to-groin delay for EVT when applicable), and center volume. Additional confounders specific to different outcomes were entered into the respective models and are detailed in the legend of Figure 1. Given the high prevalence of in-hospital stroke in the COVID-19 group, we also performed a sensitivity analysis by repeating the main multivariable analyses and including adjustments for the in-hospital stroke variable.

We expressed the results of the multivariable regression models as odds ratio and CIs. Given the potential clustering effect of patients from the same center, we included the referring center in each model as a cluster-level variable and calculated cluster-robust SEs.

To account for missing data of the covariates, we performed multiple imputations by chained equation, generating 10 imputed data sets.^19^ The rate of missing data for each variable in the registry has already been reported in the previous article.^13^ We performed analyses on each imputed data set, and then, the estimates and the SEs of the 10 imputed analyses were combined using Rubin rules.

All tests were 2 sided, and *P*<0.05 was considered significant. As this was a retrospective study, no correction for multiple-outcome testing was applied. We did not perform a power calculation because prior data estimating the expected effect of COVID-19 on the outcome of interest in revascularized stroke patients was lacking. We performed statistical analysis with R statistical software, version 4.0.3.

## RESULTS

Of 15 128 patients in the Global COVID-19 Stroke Registry, 4 were excluded because of missing data on COVID-19 symptoms, leaving 15 124 patients for the present analysis. The median age was 71.6 (interquartile range, 13.8) years, 7766 (51%) were men, 5845 patients (38.6%) were treated with IVT only, and 9279 patients (61.4%) were treated with EVT (of whom 4840 [52%] had direct EVT and 4439 [48%] had bridging).

Overall, 849 (5.6%) patients were diagnosed with COVID-19: 395 (46%) with asymptomatic (AS-COVID group) and 454 (54%) with symptomatic disease (S-COVID group). In patients with symptomatic infection, COVID-19 diagnosis was more frequent before stroke onset, while in asymptomatic patients, COVID-19 was more frequently detected within 24 hours of stroke onset (*P*<0.001; Table 1). Patients with symptomatic disease more often had in-hospital strokes compared with asymptomatic patients (46.9% versus 22.8%; *P*<0.001). Among the 213 patients with symptomatic infection and in-hospital stroke, 17% (n=37) were admitted to the intensive care unit at the time of stroke onset, while the remaining were admitted to a hospital ward.

**Table 1.**
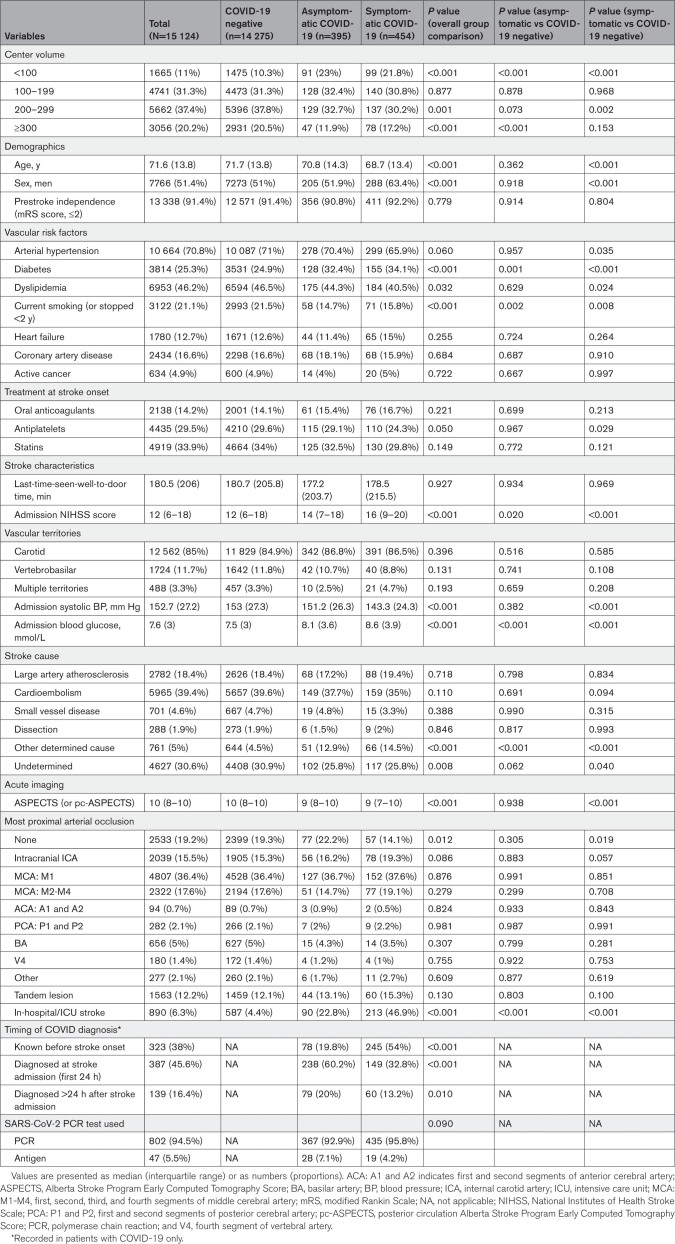
Baseline Stroke Characteristics and Imaging Data for the Overall Cohort, the COVID-19 Negative, Symptomatic, and Asymptomatic SARS-CoV-2 Infection Groups

Compared with controls, both groups of patients with AS-COVID and S-COVID had a higher prevalence of diabetes, lower prevalence of active smoking, higher baseline NIHSS score, and admission blood glucose. Patients with S-COVID were younger than controls and more frequently men and had a lower prevalence of arterial hypertension and dyslipidemia, a lower systolic blood pressure, and a lower baseline Alberta Stroke Program Early CT Score (Table 1; Tables S2 and S3 for baseline description of IVT only and EVT cohorts).

**Figure 2.**
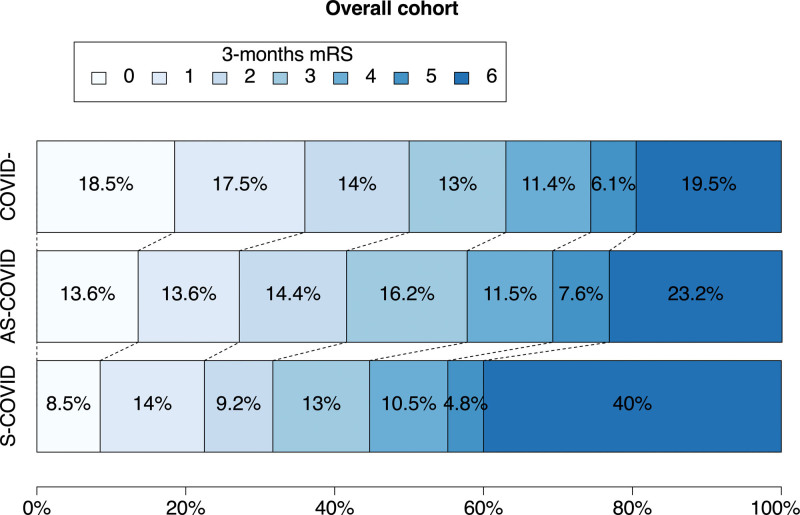
Three-month modified Rankin Scale (mRS) score distribution in asymptomatic COVID-19 (AS-COVID) and symptomatic COVID-19 (S-COVID) groups and controls.

The frequencies of each outcome in the AS-COVID, S-COVID, and control groups are shown in Table S4. The mRS score distribution of the 3 groups is displayed in the overall cohort (Figure 2) and IVT and EVT cohorts (Figure S1A and S1B).

Patients with both AS-COVID and S-COVID had a higher crude proportion of SICH (COVID-controls, 5%; AS-COVID, 7.6%; and S-COVID, 9.4%) and SICH/SSAH (COVID-controls, 6.1%; AS-COVID, 9.6%; and S-COVID, 11.7%). These differences were significant in multivariable regression analyses (Figure 1): the adjusted odds ratios (aORs) for SICH were 1.43 (95% CI, 1.03–1.99) in AS-COVID and 1.63 (95% CI, 1.14–2.32) in S-COVID; for SICH/SSAH, the aORs were 1.55 (95% CI, 1.16–2.07) in S-COVID and 1.59 (95% CI, 1.13–2.23) in S-COVID. When assessing radiological hemorrhagic transformation, we observed an increased rate of parenchymal hematomas only in the S-COVID group (11.2% versus 7.7%; *P*=0.014; Table S4). Mortality at 24 hours and 3 months was increased in patients with S-COVID (24 hours: COVID-controls, 1.3%; S-COVID, 4.8%; aOR, 2.97 [95% CI, 1.76–5.03]; *P*<0.001; 3 months: COVID-controls, 19.5%; S-COVID, 40%; aOR, 2.64 [95% CI, 2.06–3.37]; *P*<0.001; Figure 1). Disability at 3 months was worse in the AS-COVID group compared with controls (aOR, 1.25 [95% CI, 1.03–1.51]) and worst in the S-COVID group (aOR, 2.10 [95% CI, 1.75–2.53]; Figure 1), with similar effects in the analysis with 3-month good disability outcome dichotomized as mRS score ≤2 (Figure 1).

Equivalent results were obtained in the multivariable analyses performed separately on the cohorts of patients treated with IVT only and EVT (Figure S2A and S2B). The results of the sensitivity analysis including adjustment for the in-hospital stroke variable demonstrated similar findings to the main analysis, albeit with a less prominent effect (Figure S2C).

Regarding EVT-related metrics and outcomes, the S-COVID group had a higher proportion of direct EVT versus bridging compared with the COVID-negative group (61% versus 51.9%; *P*=0.004). There was a higher proportion of general anesthesia use in the S-COVID compared with COVID-negative patients (47.8% versus 35.9%; *P*<0.001; Table 2). Patients with S-COVID, but not AS-COVID, had a worse final modified Treatment in Cerebral Infarction, a lower rate of successful recanalization, a lower rate of recanalization at first pass, and a higher number of device passes compared with controls (Table 2).

**Table 2.**
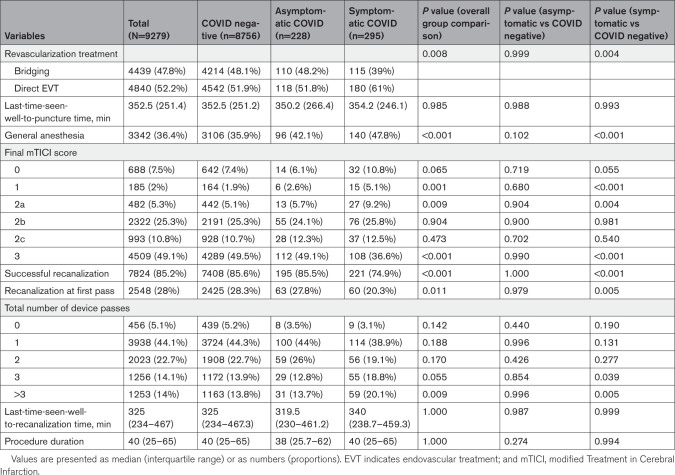
Treatment Characteristics of EVT Patients

## DISCUSSION

In this large international observational study on patients with AIS and COVID-19 undergoing acute revascularization treatment, we showed (1) the previously reported association between COVID-19 and hemorrhagic complication after revascularization stroke treatments is present for both asymptomatic and symptomatic infections; (2) 24-hour and 3-month mortalities are increased in patients with S-COVID; (3) COVID-19 is associated with a worse 3-month disability regardless of the presence of its symptoms but to a greater extent in symptomatic patients; and (4) recanalization rates were lower, and the number of EVT passes was higher only in patients with S-COVID, while no difference was observed between AS-COVID and COVID-negative controls.

The pathophysiological mechanisms associated with the risk of intracranial hemorrhage in patients with COVID-19 include systemic inflammation, endothelial dysfunction, increased blood-brain barrier permeability, hyperfibrinolysis, and coagulopathy associated with the infection.^4–6,20–24^ These alterations have mainly been observed and are more pronounced in symptomatic patients with moderate or severe disease,^25^ but they have also been described in asymptomatic infections.^26–28^ This would explain the excess of bleeding complications after revascularization treatment in patients with both S-COVID and AS-COVID.

The same biologic changes that increase the risk of bleeding in patients with COVID-19 also result in a higher clot burden, microvascular thromboinflammation, and endothelitis with subsequent impaired posttreatment reperfusion, thereby increasing the risk of stroke recurrence.^11,29^ These unfavorable effects may contribute to the greater disability outcomes observed in patients with S-COVID and, to a lesser extent, in those with asymptomatic infection.

Mortalities at 24 hours and 3 months were only increased in patients with S-COVID, suggesting that the presence of symptoms is a significant factor in the risk of death after AIS revascularization treatments. In addition to the possible contribution of biologic alterations mentioned above, the increased mortality may be explained by respiratory and systemic complications related to COVID-19, which are more common in symptomatic patients.

Less favorable EVT procedural outcomes in patients with S-COVID are likely an additional factor contributing to the worse disability and higher mortality. Worse outcomes may be explained by several potential mechanisms, including larger clots with different compositions, increased clot fragmentation leading to distal embolization, and repeated vessel reocclusion.^30–32^ We observed poorer EVT outcomes in patients with S-COVID only, while patients with asymptomatic infection did not show any differences compared with the control group. This finding is consistent with the notion that biologic alterations associated with COVID-19 correlate with disease severity and would only be severe enough in symptomatic patients to produce a measurable effect on EVT procedural outcomes. The hypothesis of a potential correlation between the symptom status of COVID-19 infection and the magnitude of its impact on AIS revascularization outcomes is also supported by the sensitivity analysis showing an attenuated effect of COVID-19 on revascularization outcomes after excluding in-hospital stroke. It is likely that patients already hospitalized for COVID-19 have more severe disease compared with those who are not.

Importantly, as previously reported in the Global COVID-19 Stroke Registry, we found no evidence of increased delay in revascularization treatment among patients with COVID-19 compared with controls. This lack of difference in treatment delays was consistent across both symptomatic and asymptomatic patients. These findings indicate that care pathways for patients with AIS were maintained regardless of COVID-19 status and symptoms and suggest that treatment delays were unlikely to be a contributing factor to the observed worse outcomes.

Our analysis has several strengths, including the large sample size with a low proportion of missing data, enabling adjustments for confounders. The inclusion of patients from 30 countries across 5 continents enhances the generalizability of our study. To our knowledge, this is the largest study to assess the safety and outcome of acute revascularization treatment in AIS in relation to AS-COVID and S-COVID.

Our study has limitations. The retrospective design cannot exclude registration bias, as academic centers may have participated more than primary stroke centers. The nonblinded assessment may have influenced reporting bias, particularly for clinical and technical outcomes. The clinical outcomes evaluated may also depend on systemic COVID-19–related complications that were not assessed in our study. Some patients with COVID-19 may have been treated outside the usual stroke care pathways, which might have affected outcomes, but we lacked this information. We cannot exclude that patients with AIS with the most severe cases of COVID-19, who would have otherwise met the criteria for IVT or EVT, did not undergo reperfusion treatment. Even with these treatments, their outcomes might have remained highly unfavorable. Hence, our results may underestimate the effect of COVID-19 on stroke outcomes. We were unable to collect data on virus variants, pandemic wave, and vaccination status of our patients, which may have influenced our results.^33^ The definition of AS-COVID was at stroke onset, and we cannot exclude that the disease became symptomatic after the stroke.

While we compared symptomatic and asymptomatic disease, our ability to assess the impact of COVID-19 severity on stroke outcomes was limited by the absence of detailed information on infection severity, aside from intensive care unit admission status. A separate analysis for this subgroup was unfeasible due to the limited number of patients admitted to the intensive care unit (n=37), precluding any meaningful statistical analysis.

Finally, our study design did not allow for conclusions about the effectiveness of revascularization treatments in patients with COVID-19 as we did not include an untreated comparison group.

## CONCLUSIONS

In conclusion, in this international retrospective cohort study, patients with AIS, AS-COVID, and S-COVID receiving revascularization treatment had higher rates of intracranial bleeding and worse short- and medium-term disabilities compared with contemporary AIS controls without COVID-19. Higher 24-hour and 3-month mortalities were observed in patients with S-COVID but not asymptomatic patients. Nevertheless, it is likely that revascularization treatments are still beneficial for both patients with AS-COVID and S-COVID, given the relatively large margin of benefit of these treatments, especially EVT, and the rather small absolute numbers of SICH in patients with AIS and COVID-19.

## ARTICLE INFORMATION

### Acknowledgments

The authors thank Melanie Price Hirt for English editing.

### Sources of Funding

The Czech stroke registry was supported by STROCZECH (Czech Stroke Research Network) within CZEch Clinical Research Infrastructure Network (CZECRIN) (No. LM2023049) funded by Czech Republic.

### Disclosures

Dr Nguyen is on the Advisory Board of Idorsia. Dr Michel received research grants from the Swiss National Science Foundation, the Swiss Heart Foundation, and the University of Lausanne. The other authors report no conflicts. Global COVID-19 Stroke Registry: R. Herzig received research grants from the Ministry of Health of the Czech Republic (Grant number DRO – UHHK 00179906) and Charles University, Czech Republic (Cooperatio Program). C. Nolte received research grants from the German Ministry of Research and Education, the German Center for Neurodegenerative Diseases, and the German Center for Cardiovascular Research and is a speaker and advisory for Abbott, Alexion, Bayer, Boehringer Ingelheim, Bristol-Myers Squibb, Daiichi Sankyo, and Pfizer Pharma. S. Tjoumakaris is on Advisory Medtronic and MicroVention. J. Min is on Advisory Medtronic and Abbott. M.-A. Khan received research grants from National Institutes of Health, Spectrum Health-Michigan State University Research Alliance, and Genentech. A. Zini received consultation and travel expenses from Alexion-AstraZeneca, CSL Behring, and Boehringer Ingelheim and is on the steering committee of the OCEANIC-STROKE trial (A Study to Test Asundexian to Prevent a Clot-Related Stroke in Participants After an Acute Ischemic Stroke or High-Risk TIA/Mini-Stroke). A. Slowik and P. Wrona received research grant from European Research Area Network (ERA-NET) - Network of European Funding for Neuroscience Research (NEURON)/21/2020 Identification and clinical validation of biomarkers for long-term outcome after cerebral ischaemia (iBioStroke).

### Supplemental Material

Tables S1–S4

Figures S1–S2

## APPENDIX

Global COVID-19 Stroke Registry: Roman Herzig (Neurology, Comprehensive Stroke Center, Charles University Faculty of Medicine and University Hospital, Hradec Králové, Czech Republic), Anna Członkowksa (2nd Department of Neurology, Institute of Psychiatry and Neurology, Warsaw, Poland), Jelle Demeestere (Neurology, Leuven University Hospital, Belgium), Ossama Yassin Mansour (Alexandria University Hospitals and Affiliated Stroke Network, Egypt), Georgios Georgiopoulos (School of Biomedical Engineering and Imaging Sciences, St Thomas Hospital, King’s College London, United Kingdom; Clinical Therapeutics, National and Kapodistrian University of Athens, Greece), Raul G. Nogueira (Neurology, Neurosurgery, UPMC), Alexander Salerno (Stroke Center, Neurology Service, Department of Neurological Sciences, Lausanne University Hospital and University of Lausanne, Switzerland), Susanne Wegener (Neurology, University Hospital of Zürich, Switzerland), Philipp Baumgartner (Neurology, University Hospital of Zürich, Switzerland), Carlo W. Cereda (Neurology Clinic, Stroke Center, Neurocenter of Southern Switzerland, Ente Ospedaliero Cantonale, Lugano, Switzerland), Giovanni Bianco (Neurology Clinic, Stroke Center, Neurocenter of Southern Switzerland, Ente Ospedaliero Cantonale, Lugano, Switzerland), Morin Beyeler (Stroke Center, Neurology, Inselspital, Bern University Hospital, University of Bern, Switzerland), Marcel Arnold (Stroke Center, Neurology, Inselspital, Bern University Hospital, University of Bern, Switzerland), Emmanuel Carrera (Stroke Center, Geneva University Hospital, Switzerland), Paolo Machi (Neuroradiology, Geneva University Hospital, Switzerland), Valerian Altersberger (Stroke Center, University Hospital Basel, University of Basel, Switzerland), Leo Bonati (Stroke Center, University Hospital Basel, University of Basel, Switzerland), Henrik Gensicke (Stroke Center, University Hospital Basel, University of Basel, Switzerland), Manuel Bolognese (Stroke Center, Kantonsspital Lucerne, Switzerland), Nils Peters (Stroke Center, Hirslanden Hospital, Zürich, Switzerland), Stephan Wetzel (Stroke Center, Hirslanden Hospital, Zürich, Switzerland), Marta Magriço (Stroke Center, Neurology Service, Department of Neurological Sciences, Lausanne University Hospital and University of Lausanne, Switzerland), João Nuno Ramos (Neuroradiology, Hospital de Egas Moniz, Centro Hospitalar Lisboa Ocidental, Portugal), João Sargento-Freitas (Neurology, Centro Hospitalar Universitário de Coimbra, Portugal), Rita Machado (Neurology, Centro Hospitalar Universitário de Coimbra, Portugal), Carolina Maia (Neuroradiology, Centro Hospitalar Universitário de Coimbra, Portugal), Egídio Machado (Stroke Unit, Hospital de São José, Centro Hospitalar Universitário Lisboa Central, Portugal), Ana Paiva-Nunes (Stroke Unit, Neurology, Hospital de Santa Maria, Centro Hospitalar Universitário Lisboa Norte, Portugal), Patrícia Ferreira (Stroke Unit, Neurology, Hospital de Santa Maria, Centro Hospitalar Universitário Lisboa Norte, Portugal), Teresa Pinho-e-Melo (Neuroradiology, Hospital de Santa Maria, Centro Hospitalar Universitário Lisboa Norte, Portugal), Mariana Carvalho-Dias (Neuroradiology, Hospital de Santa Maria, Centro Hospitalar Universitário Lisboa Norte, Portugal), André Paula (Neuroradiology, Hospital de Santa Maria, Centro Hospitalar Universitário Lisboa Norte, Portugal), Manuel Alberto Correia (Neuroradiology, Hospital de Santa Maria, Centro Hospitalar Universitário Lisboa Norte, Portugal), Pedro Castro (Neurology, Centro Hospitalar Universitário São João, Porto, Portugal), Elsa Azevedo (Neurology, Centro Hospitalar Universitário São João, Porto, Portugal), Luís Albuquerque (Neuroradiology, Centro Hospitalar Universitário São João, Porto, Portugal), José Nuno-Alves (Neuroradiology, Centro Hospitalar Universitário São João, Porto, Portugal), Joana Ferreira-Pinto (Neurology, Hospital de Braga, Portugal), Torcato Meira (Neuroradiology, Hospital de Braga, Portugal), Liliana Pereira (Neurology, Hospital Garcia de Orta, Almada, Portugal), Miguel Rodrigues (Neurology, Hospital Garcia de Orta, Almada, Portugal), André Araújo (Neuroradiology, Centro Hospitalar de Vila Nova de Gaia/Espinho, Portugal), Marta Rodrigues (Neuroradiology, Centro Hospitalar de Vila Nova de Gaia/Espinho, Portugal), Mariana Rocha (Neurology, Centro Hospitalar de Vila Nova de Gaia/Espinho, Portugal), Ângelo Pereira-Fonseca (Neurology, Unidade Local de Saúde de Matosinhos, Portugal), Luís Ribeiro (Neurology, Unidade Local de Saúde de Matosinhos, Portugal), Ricardo Varela (Neurology, Centro Hospitalar Universitário do Porto, Portugal), Manuel Cappellari (Stroke Unit, Azienda Ospedaliera Universitaria Integrata, Verona, Italy), Cecilia Zivelonghi (Stroke Unit, Azienda Ospedaliera Universitaria Integrata, Verona, Italy), Giulia Sajeva (Stroke Unit, Azienda Ospedaliera Universitaria Integrata, Verona, Italy), Andrea Zini (IRCCS Istituto delle Scienze Neurologiche di Bologna, Neurology and Stroke Center, Maggiore Hospital, Italy), Mauro Gentile (IRCCS Istituto delle Scienze Neurologiche di Bologna, Neurology and Stroke Center, Maggiore Hospital, Italy), Stefano Forlivesi (IRCCS Istituto delle Scienze Neurologiche di Bologna, Neurology and Stroke Center, Maggiore Hospital, Italy), Ludovica Migliaccio (IRCCS Istituto delle Scienze Neurologiche di Bologna, Neurology and Stroke Center, Maggiore Hospital, Italy), Maria Sessa (Neurology, ASST Papa Giovanni XXIII, Bergamo, Italy), Alessandro Pezzini (Department of Medicine and Surgery, University of Parma, Italy; Stroke Care Program, Department of Emergency, Parma University Hospital, Italy; Clinical and Experimental Sciences, Neurology Clinic, University of Brescia, Italy), Davide Sangalli (Neurology and Stroke Unit, Azienda Socio Sanitaria Territoriale, Lecco, Italy), Marialuisa Zedde (Neurology Unit, Stroke Unit, Azienda Unità Sanitaria-IRCCS di Reggio Emilia, Italy), Rosario Pascarella (Neuroradiology Unit, Azienda Unità Sanitaria-IRCCS di Reggio Emilia, Italy), Susanna Diamanti (Neurology, San Gerardo Hospital, Department of Medicine and Surgery and Milan Center for Neuroscience, University of Milano Bicocca, Monza, Italy), Simone Beretta (Neurology, San Gerardo Hospital, Department of Medicine and Surgery and Milan Center for Neuroscience, University of Milano Bicocca, Monza, Italy), Ghil Schwarz (Stroke Unit, Fondazione IRCCS Ca’ Granda Ospedale Maggiore Policlinico, Milan, Italy), Giovanni Frisullo (Neurology, Policlinico Universitario Agostino Gemelli, Rome, Italy), Simona Marcheselli (Emergency Neurology and Stroke Unit, IRCCS Humanitas Clinical and Research Center, Rozzano, Italy), Pierre Seners (Neurology, Hôpital Fondation Adolphe De Rothschild, Paris, France), Candice Sabben (Neurology, Hôpital Fondation Adolphe De Rothschild, Paris, France), Simon Escalard (Interventional Neuroradiology, Hôpital Fondation Adolphe De Rothschild, Paris, France), Michel Piotin (Interventional Neuroradiology, Hôpital Fondation Adolphe De Rothschild, Paris, France), Benjamin Maier (Interventional Neuroradiology, Hôpital Fondation Adolphe De Rothschild, Paris, France), Guillaume Charbonnier (Interventional Neuroradiology, Center Hospitalier Régional Universitaire, Hôpital Jean Minjoz, Besançon, France), Fabrice Vuillier (Interventional Neuroradiology, Center Hospitalier Régional Universitaire, Hôpital Jean Minjoz, Besançon, France), Loic Legris (University Grenoble Alpes, Stroke Unit, France), Pauline Cuisenier (University Grenoble Alpes, Stroke Unit, France), Francesca R. Vodret (University Grenoble Alpes, Stroke Unit, France), Gaultier Marnat (University of Bordeaux, Bordeaux University Hospital, Stroke Unit, France), Jean-Sebastien Liegey (University of Bordeaux, Bordeaux University Hospital, Stroke Unit, France), Igor Sibon (University of Bordeaux, Bordeaux University Hospital, Stroke Unit, France), Fabian Flottmann (Diagnostic and Interventional Neuroradiology, University Medical Center-Hamburg-Eppendorf, Germany), Gabriel Broocks (Diagnostic and Interventional Neuroradiology, University Medical Center-Hamburg-Eppendorf, Germany), Nils-Ole Gloyer (Diagnostic and Interventional Neuroradiology, University Medical Center-Hamburg-Eppendorf, Germany), Ferdinand O. Bohmann (Neurology, University Hospital Frankfurt, Goethe University, Germany), Jan Hendrik Schaefer (Neurology, University Hospital Frankfurt, Goethe University, Germany), Christian H. Nolte (Neurology, Center for Stroke Research, Berlin Institute of Health, Charité-Universitätsmedizin Berlin, Germany), Heinrich Audebert (Neurology, Center for Stroke Research, Berlin Institute of Health, Charité-Universitätsmedizin Berlin, Germany), Eberhard Siebert (Neuroradiology, Charité-Universitätsmedizin Berlin, Germany), Marek Sykora (Neurology, St. John’s Hospital, Vienna, Austria), Wilfried Lang (Neurology, St. John’s Hospital, Vienna, Austria), Julia Ferrari (Neurology, St. John’s Hospital, Vienna, Austria), Lukas Mayer-Suess (Neurology, Medical University of Innsbruck, Austria), Michael Knoflach (Neurology, Medical University of Innsbruck, Austria), Elke-Ruth Gizewski (Neuroradiology, Medical University of Innsbruck, Austria), Jeffrey Stolp (Neurology, Amsterdam University Medical Centers, Amsterdam Neuroscience, University of Amsterdam, the Netherlands), Lotte J. Stolze (Neurology, Amsterdam University Medical Centers, Amsterdam Neuroscience, University of Amsterdam, the Netherlands), Jonathan M. Coutinho (Neurology, Amsterdam University Medical Centers, Amsterdam Neuroscience, University of Amsterdam, the Netherlands), Paul J. Nederkoorn (Neurology, Amsterdam University Medical Centers, Amsterdam Neuroscience, University of Amsterdam, the Netherlands), Ido van-den-Wijngaard (Neurology, Haaglanden Medical Center, Hague; Radiology, Leiden University Medical Center, the Netherlands), Joke de Meris (Neurology, Haaglanden Medical Center, Hague; Radiology, Leiden University Medical Center, the Netherlands), Robin Lemmens (Neurology, Leuven University Hospital, Belgium), Sylvie De Raedt (Neurology, Universitair Ziekenhuis Brussel, Center for Neurosciences, Vrije Universiteit Brussel, Belgium), Fenne Vandervorst (Neurology, Universitair Ziekenhuis Brussel, Center for Neurosciences, Vrije Universiteit Brussel, Belgium), Matthieu Pierre Rutgers (Neurology, Stroke Unit, Europe Hospitals, Brussels, Belgium), Antoine Guilmot (Neurology, Stroke Unit, Europe Hospitals, Brussels, Belgium), Anne Dusart (Neurology, Centre Hospitalier Universitaire de Charleroi, Belgium), Flavio Bellante (Neurology, Centre Hospitalier Universitaire de Charleroi, Belgium), Patricia Calleja-Castaño (Neurology and Stroke Center, Hospital Universitario 12 de Octubre, Instituto de Investigación Hospital 12 de Octubre, Madrid, Spain), Fernando Ostos (Neurology and Stroke Center, Hospital Universitario 12 de Octubre, Instituto de Investigación Hospital 12 de Octubre, Madrid, Spain), Guillermo Gonzalez-Ortega (Neurology and Stroke Center, Hospital Universitario 12 de Octubre, Instituto de Investigación Hospital 12 de Octubre, Madrid, Spain), Paloma Martín-Jiménez (Neurology and Stroke Center, Hospital Universitario 12 de Octubre, Instituto de Investigación Hospital 12 de Octubre, Madrid, Spain), Sebastian García-Madrona (Neurology, Ramón y Cajal University Hospital, Madrid, Spain), Antonio Cruz-Culebras (Neurology, Ramón y Cajal University Hospital, Madrid, Spain), Rocio Vera (Neurology, Ramón y Cajal University Hospital, Madrid, Spain), Maria-Consuelo Matute (Neurology, Ramón y Cajal University Hospital, Madrid, Spain), Blanca Fuentes (Neurology and Stroke Center, La Paz University Hospital, Institute for Health Research-IdiPAZ, Madrid, Spain), María Alonso-de-Leciñana (Neurology and Stroke Center, La Paz University Hospital, Institute for Health Research-IdiPAZ, Madrid, Spain), Ricardo Rigual (Neurology and Stroke Center, La Paz University Hospital, Institute for Health Research-IdiPAZ, Madrid, Spain), Exuperio Díez-Tejedor (Neurology and Stroke Center, La Paz University Hospital, Institute for Health Research-IdiPAZ, Madrid, Spain), Soledad Pérez-Sánchez (Neurology, Hospital Universitario Virgen Macarena, Seville, Spain), Joan Montaner (Neurology, Hospital Universitario Virgen Macarena, Seville, Spain), Fernando Díaz-Otero (Stroke Center, Hospital General Universitario Gregorio Marañón, Madrid, Spain), Natalia Perez de la Ossa (Stroke Unit, Germans Trias i Pujol Hospital, Barcelona, Spain), Belén Flores-Pina (Stroke Unit, Germans Trias i Pujol Hospital, Barcelona, Spain), Lucia Muñoz-Narbona (Stroke Unit, Germans Trias i Pujol Hospital, Barcelona, Spain), Angel Chamorro (Neurology, Comprehensive Stroke Center, Hospital Clinic From Barcelona, Spain), Alejandro Rodríguez-Vázquez (Neurology, Comprehensive Stroke Center, Hospital Clinic From Barcelona, Spain), Arturo Renú (Neurology, Comprehensive Stroke Center, Hospital Clinic From Barcelona, Spain), Oscar Ayo-Martin (Neurology, Complejo Hospitalario Universitario de Albacete, Spain), Francisco Hernandez-Fernandez (Neurology, Complejo Hospitalario Universitario de Albacete, Spain), Tomas Segura (Neurology, Complejo Hospitalario Universitario de Albacete, Spain), Herbert Tejada-Meza (Stroke Unit, Neurology, and Interventional Neuroradiology Unit, Radiology, Hospital Universitario Miguel Servet, Spain), Thant Hlaing (Stroke and Geriatric Medicine, Aintree University Hospital, Liverpool, United Kingdom), Isaiah See (Comprehensive Stroke Service, University College London Hospitals NHS Foundation Trust and Stroke Research Centre, University College London, United Kingdom), Robert Simister (Comprehensive Stroke Service, University College London Hospitals NHS Foundation Trust and Stroke Research Centre, University College London, United Kingdom), David J. Werring (University College London, Queen Square Institute of Neurology, United Kingdom), Espen Saxhaug Kristoffersen (Neurology, Akershus University Hospital, Lørenskog and Department of General Practice, University of Oslo, Norway), Annika Nordanstig (Clinical Neuroscience, Institute of Neuroscience and Physiology, Sahlgrenska Academy at University of Gothenburg; Neurology, Sahlgrenska University Hospital, Region Västra Götaland, Sweden), Katarina Jood (Clinical Neuroscience, Institute of Neuroscience and Physiology, Sahlgrenska Academy at University of Gothenburg; Neurology, Sahlgrenska University Hospital, Region Västra Götaland, Sweden), Alexandros Rentzos (Radiology, Institute of Clinical Sciences, Sahlgrenska Academy at the University of Gothenburg; Interventional and Diagnostic Neuroradiology, Sahlgrenska University Hospital, Region Västra Götaland, Sweden), Libor Šimůnek (Neurology, Comprehensive Stroke Center, Charles University Faculty of Medicine and University Hospital, Hradec Králové, Czech Republic), Dagmar Krajíčková (Neurology, Comprehensive Stroke Center, Charles University Faculty of Medicine and University Hospital, Hradec Králové, Czech Republic), Antonín Krajina (Radiology, Comprehensive Stroke Center, Charles University Faculty of Medicine and University Hospital, Hradec Králové, Czech Republic), Robert Mikulík (Radiology, Comprehensive Stroke Center, Charles University Faculty of Medicine and University Hospital, Hradec Králové, Czech Republic), Martina Cviková (Radiology, Comprehensive Stroke Center, Charles University Faculty of Medicine and University Hospital, Hradec Králové, Czech Republic), Jan Vinklárek (International Clinical Research Center and Department of Neurology, St. Anne’s University Hospital, Faculty of Medicine at Masaryk University, Brno, Czech Republic), David Školoudík (Neurology, University Hospital Ostrava, Czech Republic), Martin Roubec (Neurology, University Hospital Ostrava, Czech Republic), Eva Hurtikova (Neurology, University Hospital Ostrava, Czech Republic), Rostislav Hrubý (Neurology, České Budějovice Hospital, Czech Republic), Svatopluk Ostry (Neurology, České Budějovice Hospital, Czech Republic), Ondrej Skoda (Neurology, Jihlava Hospital, Czech Republic), Marek Pernicka (Neurology, Jihlava Hospital, Czech Republic), Lubomír Kočí (Neurocenter, Regional Hospital Liberec, Czech Republic), Zuzana Eichlová (Neurocenter, Regional Hospital Liberec, Czech Republic), Martin Jíra (Neurocenter, Regional Hospital Liberec, Czech Republic), Martin Kovář (Cerebrovascular Center, Na Homolce Hospital, Prague, Czech Republic), Michal Panský (Cerebrovascular Center, Na Homolce Hospital, Prague, Czech Republic), Pavel Mencl (Cerebrovascular Center, Na Homolce Hospital, Prague, Czech Republic), Hana Paloušková (Neurology, Karviná Miners Hospital, Inc, Czech Republic), Aleš Tomek (Cerebrovascular Center, University Hospital in Motol, Prague, Czech Republic), Petr Janský (Cerebrovascular Center, University Hospital in Motol, Prague, Czech Republic), Anna Olšerová (Cerebrovascular Center, University Hospital in Motol, Prague, Czech Republic), Martin Šrámek (Cerebrovascular Center, Central Military Hospital, Prague, Czech Republic), Roman Havlíček (Cerebrovascular Center, Central Military Hospital, Prague, Czech Republic), Petr Malý (Cerebrovascular Center, Central Military Hospital, Prague, Czech Republic), Lukáš Trakal (Cerebrovascular Center, Central Military Hospital, Prague, Czech Republic), Jan Fiksa (Cerebrovascular Center, General University Hospital, Prague, Czech Republic), Matěj Slovák (Cerebrovascular Center, General University Hospital, Prague, Czech Republic), Michał Karliński (2nd Department of Neurology, Institute of Psychiatry and Neurology, Warsaw, Poland), Maciej Nowak (2nd Department of Neurology, Institute of Psychiatry and Neurology, Warsaw, Poland), Halina Sienkiewicz-Jarosz (1st Department of Neurology, Institute of Psychiatry and Neurology, Warsaw, Poland), Anna Bochynska (1st Department of Neurology, Institute of Psychiatry and Neurology, Warsaw, Poland), Pawel Wrona (Neurology, University Hospital, Jagiellonian University, Kraków, Poland), Tomasz Homa (Neurology, University Hospital, Jagiellonian University, Kraków, Poland), Katarzyna Sawczynska (Neurology, University Hospital, Jagiellonian University, Kraków, Poland), Agnieszka Slowik (Neurology, University Hospital, Jagiellonian University, Kraków, Poland), Ewa Wlodarczyk (Neurology, University Hospital, Jagiellonian University, Kraków, Poland), Marcin Wiącek (Neurology, Institute of Medical Sciences, Medical College of Rzeszów University, Rzeszów, Poland), Izabella Tomaszewska-Lampart (Neurology, Institute of Medical Sciences, Medical College of Rzeszów University, Rzeszów, Poland), Bartosz Sieczkowski (Neurology, Institute of Medical Sciences, Medical College of Rzeszów University, Rzeszów, Poland), Halina Bartosik-Psujek (Neurology, Institute of Medical Sciences, Medical College of Rzeszów University, Rzeszów, Poland), Marta Bilik (Neurology and Stroke, St. John Paul II Western Hospital, Grodzisk Mazowiecki, Poland), Anna Bandzarewicz (Neurology and Stroke, St. John Paul II Western Hospital, Grodzisk Mazowiecki, Poland), Malgorzata Dorobek (Neurology, National Medical Institute of the Ministry of Interior and Administration, Warsaw, Poland), Justyna Zielińska-Turek (Neurology, National Medical Institute of the Ministry of Interior and Administration, Warsaw, Poland), Marta Nowakowska-Kotas (Neurology, Wroclaw Medical University, Poland), Krystian Obara (Neurology, Wroclaw Medical University, Poland), Paweł Urbanowski (Neurology, Wroclaw Medical University, Poland), Sławomir Budrewicz (Neurology, Wroclaw Medical University, Poland), Maciej Guziński (Radiology, Wroclaw Medical University, Poland), Milena Świtońska (Neurosurgery and Neurology, Nicolaus Copernicus University in Torun Ludwik Rydygier Collegium Medicum, Bydgoszcz, Poland), Iwona Rutkowska (Stroke Intervention Center, Neurosurgery and Neurology, Jan Biziel University Hospital, Bydgoszcz, Poland), Paulina Sobieszak-Skura (Stroke Intervention Center, Neurosurgery and Neurology, Jan Biziel University Hospital, Bydgoszcz, Poland), Beata Łabuz-Roszak (Neurology, Institute of Medical Sciences, University of Opole, Poland), Aleksander Dębiec (Clinic of Neurology, Military Institute of Medicine, Warsaw, Poland), Jacek Staszewski (Clinic of Neurology, Military Institute of Medicine, Warsaw, Poland), Adam Stępień (Clinic of Neurology, Military Institute of Medicine, Warsaw, Poland), Jacek Zwiernik (Neurology, University of Warmia and Mazury, Olsztyn, Poland), Grzegorz Wasilewski (Radiology, Provincial Specialist Hospital, Olsztyn, Poland), Cristina Tiu (Neurology, University Emergency Hospital Bucharest, University of Medicine and Pharmacy “Carol Davila,” Romania), Elena-Oana Terecoasă (Neurology, University Emergency Hospital Bucharest, University of Medicine and Pharmacy “Carol Davila,” Romania), Razvan-Alexandru Radu (Neurology, University Emergency Hospital Bucharest, University of Medicine and Pharmacy “Carol Davila,” Romania), Anca Negrila (Neurology, University Emergency Hospital Bucharest, University of Medicine and Pharmacy “Carol Davila,” Romania), Bogdan Dorobat (Radiology, University Emergency Hospital Bucharest, Romania), Cristina Panea (Neurology and Stroke Unit, Elias University Emergency Hospital, University of Medicine and Pharmacy “Carol Davila,” Bucharest, Romania), Vlad Tiu (Neurology and Stroke Unit, Elias University Emergency Hospital, University of Medicine and Pharmacy “Carol Davila,” Bucharest, Romania), Simona Petrescu (Neurology and Stroke Unit, Elias University Emergency Hospital, University of Medicine and Pharmacy “Carol Davila,” Bucharest, Romania), Atilla Özcan-Özdemir (Neurology, Eskisehir Osmangazi University, Turkey), Mostafa Mahmoud (Ain Shams University Affiliated Saudi German Hospital, Cairo, Egypt), Hussam El-Samahy (Ain Shams University Affiliated Saudi German Hospital, Cairo, Egypt), Hazem Abdelkhalek (Neuropsychiatry Department, Tanta University, Egypt), Jasem Al-Hashel (Neurology, Ibn Sina Hospital, Kuwait), Ismail Ibrahim Ismail (Neurology, Ibn Sina Hospital, Kuwait), Athari Salmeen (Neurology, Jaber Al-Ahmad Hospital, Kuwait), Abdoreza Ghoreishi (Neurology, School of Medicine, Zanjan University of Medical Sciences, Iran), Sergiu Sabetay (Stroke Unit, Neurology, Hillel Yaffe Medical Center, Hadera, Israel), Hana Gross (Neurology, Radiology, Boston Medical Center, MA), Piers Klein (Neurology, Radiology, Boston Medical Center, MA), Mohamad Abdalkader (Neurology, Radiology, Boston Medical Center, MA), Pascal Jabbour (Neurosurgery, Thomas Jefferson University Hospital, Philadelphia, PA), Kareem El Naamani (Neurosurgery, Thomas Jefferson University Hospital, Philadelphia, PA), Stavropoula Tjoumakaris (Neurosurgery, Thomas Jefferson University Hospital, Philadelphia, PA), Rawad Abbas (Neurosurgery, Thomas Jefferson University Hospital, Philadelphia, PA), Ghada-A. Mohamed (Neurology, Neurosurgery, UPMC; Neurology, Henry Ford Hospital, Detroit, MI), Alex Chebl (Neurology, Henry Ford Hospital, Detroit, MI), Jiangyong Min (Neurosciences, Spectrum Health and Michigan State University, East Lansing), Majesta Hovingh (Neurosciences, Spectrum Health and Michigan State University, East Lansing), Jenny Tsai (Neurosciences, Spectrum Health and Michigan State University, East Lansing), Muhib-A. Khan (Neurosciences, Spectrum Health and Michigan State University, East Lansing), Krishna Nalleballe (Neurology, University of Arkansas for Medical Sciences, Little Rock), Sanjeeva Onteddu (Neurology, University of Arkansas for Medical Sciences, Little Rock), Hesham E. Masoud (Neurology, Upstate University Hospital, Syracuse, NY), Mina Michael (Neurology, Upstate University Hospital, Syracuse, NY), Navreet Kaur (Neurology, Upstate University Hospital, Syracuse, NY), Laith Maali (Neurology, University of Kansas Medical Center, Lawrence, KS), Michael Abraham (Neurology, University of Kansas Medical Center, Lawrence, KS), Priyank Khandelwal (Endovascular Neurological Surgery and Neurology, Rutgers, The State University of New Jersey, Newark), Ivo Bach (Endovascular Neurological Surgery and Neurology, Rutgers, The State University of New Jersey, Newark), Melody Ong (Endovascular Neurological Surgery and Neurology, Rutgers, The State University of New Jersey, Newark), Denis Babici (Endovascular Neurological Surgery and Neurology, Rutgers, The State University of New Jersey, Newark), Ayaz-M. Khawaja (Neurology, Wayne State University, Detroit Medical Center, MI), Maryam Hakemi (Neurology, Wayne State University, Detroit Medical Center, MI), Kumar Rajamani (Neurology, Wayne State University, Detroit Medical Center, MI), Vanessa Cano-Nigenda (Stroke Clinic, Instituto Nacional de Neurologia y Neurocirugia Manuel Velasco Suarez, Mexico City, Mexico), Antonio Arauz (Stroke Clinic, Instituto Nacional de Neurologia y Neurocirugia Manuel Velasco Suarez, Mexico City, Mexico), Pablo Amaya (Neurology, Fundación Valle del Lili, Cali, Colombia), Natalia Llanos (Centro de Investigaciones Clínicas, Fundación Valle del Lili, Cali, Colombia), Akemi Arango (Centro de Investigaciones Clínicas, Fundación Valle del Lili, Cali, Colombia), Miguel A. Vences (Neurology, Hospital Nacional Edgardo Rebagliati Martins, EsSalud, Lima, Peru), José-Domingo Barrientos (Hospital General San Juan de Dios, Guatemala City, Guatemala), Rayllene Caetano (Neurology, Hospital Nossa Senhora da Conceição Hospital, Porto Alegre, Brazil), Rodrigo Targa (Neurology, Hospital Nossa Senhora da Conceição Hospital, Porto Alegre, Brazil), Sergio Scollo (Ramos Mejía Hospital, Stroke Unit, Buenos Aires, Argentina), Patrick Yalung (St. Luke’s Medical Center, Global City, Philippines), Shashank Nagendra (Neurology, Grant Medical College and Sir JJ Hospital, Mumbai, India), Abhijit Gaikwad (Neurology, Grant Medical College and Sir JJ Hospital, Mumbai, India), Kwon-Duk Seo (Neurology, National Health Insurance Service Ilsan Hospital, Goyang, Korea).

## Supplementary Material


