# Supplementary material for: Effect of Asymptomatic and Symptomatic COVID-19 on Acute Ischemic Stroke Revascularization Outcomes
Source: Stroke. 2023 Dec 12;55(1):78–88. doi: 10.1161/STROKEAHA.123.043899 (PMC10734790; doi:10.1161/STROKEAHA.123.043899)
Supplement: Supplementary file 1 [file str-55-078-s001.pdf]

SUPPLEMENTAL MATERIAL

Table S1.

WHO Clinical criteria for suspected case of SARS-CoV-2 infection.
Acute onset of fever AND cough<br>OR<br>Acute onset of ANY THREE OR MORE of the following signs or symptoms:<br>- fever<br>- cough<br>- general weakness/fatigue<br>- headache<br>- myalgia<br>- sore throat<br>- coryza<br>- dyspnea<br>- nausea/diarrhea/anorexia<br>OR<br>A patient with severe acute respiratory illness (SARI): acute respiratory infection with history of fever or measured fever of ≥38 °C AND cough
Adapted from: WHO COVID-19: Case Definitions. https://www.who.int/publications/i/item/who-2019-nCoV-surveillanceguidance-2020.7. under the terms and conditions of the Creative Commons Attribution (CC BY-NC-SA) license (https://creativecommons.org/licenses/by-nc-sa/4.0/#).

Table S2. Baseline stroke characteristics and imaging data for the IVT-only cohort, the COVID-19 negative, symptomatic, and asymptomatic SARS-CoV-2 infection groups.

Variables	Total (n=5845)	COVID negative (n=5519)	Asymptomatic (n=167)	Symptomatic (n=159)	P-value (Overall group comparison)	P-value (asymptomatic vs. COVID negative)	P-value (symptomatic vs. COVID negative)
Center volume							
< 100	924 (15.8%)	823 (14.9%)	53 (31.7%)	48 (30.2%)	0.000	0.000	0.000
100-199	2045 (35%)	1950 (35.3%)	53 (31.7%)	42 (26.4%)	0.045	0.560	0.040
200-299	1985 (34%)	1879 (34%)	51 (30.5%)	55 (34.6%)	0.632	0.572	0.987
>=300	891 (15.2%)	867 (15.7%)	10 (6%)	14 (8.8%)	0.000	0.001	0.033
Demographics					0.000		
Age	72.1 (14)	72.2 (14)	72.1 (13.6)	69.5 (13.7)	0.056	0.993	0.033
Sex	3221 (55.1%)	3032 (55%)	91 (54.5%)	98 (61.6%)	0.244	0.991	0.180
Pre-stroke independency (mRS ≤2)	4985 (88.2%)	4702 (88.3%)	144 (86.8%)	139 (88.5%)	0.827	0.793	0.994
Vascular risk factors					0.000		
Arterial hypertension	4232 (72.7%)	4002 (72.8%)	123 (73.7%)	107 (67.3%)	0.296	0.962	0.235
Diabetes mellitus	1537 (26.4%)	1429 (26%)	55 (32.9%)	53 (33.3%)	0.018	0.090	0.077
Dyslipidemia	2728 (46.9%)	2585 (47.1%)	83 (49.7%)	60 (37.7%)	0.051	0.755	0.039
Current smoking (or stopped < 2 years)	1168 (20.3%)	1133 (20.9%)	18 (10.8%)	17 (10.9%)	0.000	0.003	0.004
Heart failure	475 (8.8%)	446 (8.8%)	12 (7.2%)	17 (11%)	0.493	0.732	0.575
Coronary artery disease	941 (16.8%)	891 (16.8%)	26 (16.8%)	24 (16.6%)	0.996	1.000	0.995
Active cancer	215 (4.4%)	204 (4.4%)	5 (3.5%)	6 (4.2%)	0.841	0.809	0.989
Treatment at stroke onset					0.000		
Oral anticoagulants	353 (6.1%)	336 (6.1%)	8 (4.8%)	9 (5.7%)	0.760	0.729	0.965
Antiplatelets	2090 (35.9%)	1989 (36.2%)	53 (31.7%)	48 (30.2%)	0.156	0.418	0.225
Statins	2016 (34.6%)	1911 (34.8%)	63 (37.7%)	42 (26.6%)	0.071	0.676	0.064
Stroke characteristics					0.000		
Last-Time-Seen-Well-to-door delay (minutes)	131.6 (129.7)	131.5 (129.4)	138.6 (134.9)	125.3 (136.8)	0.659	0.748	0.801
NIHSS admission	7 (4-12)	6 (4-11)	8 (5-14)	10 (5-17.3)	0.000	0.034	0.000
Vascular territories							

Variables	Total (n=5845)	COVID negative (n=5519)	Asymptomatic (n=167)	Symptomatic (n=159)	P-value (Overall group comparison)	P-value (asymptomatic vs. COVID negative)	P-value (symptomatic vs. COVID negative)
Carotid	4382 (78.8%)	4118 (78.7%)	134 (80.7%)	130 (81.8%)	0.532	0.772	0.573
Vertebrobasilar	908 (16.3%)	863 (16.5%)	25 (15.1%)	20 (12.6%)	0.382	0.859	0.343
Multiple territories	270 (4.9%)	254 (4.8%)	7 (4.2%)	9 (5.7%)	0.831	0.915	0.871
Admission systolic BP (mmHG)	157.7 (28)	158.1 (28)	155.9 (26.9)	146.3 (26.3)	0.000	0.550	0.000
Admission blood glucose (mmol/l)	7.5 (3.2)	7.5 (3.1)	8.4 (3.8)	8.7 (4.5)	0.000	0.000	0.000
Stroke etiology							
Large artery atherosclerosis	952 (16.3%)	902 (16.3%)	31 (18.6%)	19 (11.9%)	0.242	0.691	0.259
Cardioembolism	1659 (28.4%)	1571 (28.5%)	44 (26.4%)	44 (27.7%)	0.819	0.797	0.970
Small vessels disease	671 (11.5%)	638 (11.6%)	19 (11.4%)	14 (8.8%)	0.561	0.997	0.485
Dissection	77 (1.3%)	72 (1.3%)	2 (1.2%)	3 (1.9%)	0.810	0.991	0.775
Other determined cause	346 (5.9%)	296 (5.4%)	20 (12%)	30 (18.9%)	0.000	0.001	0.000
Undetermined	2140 (36.6%)	2040 (37%)	51 (30.5%)	49 (30.8%)	0.073	0.171	0.213
Acute imaging					0.000		
ASPECTS (or pc-ASPECTS)	10 (9-10)	10 (9-10)	10 (9-10)	10 (8-10)	0.000	0.030	0.000
Most proximal arterial occlusion							
None	2461 (60.5%)	2329 (60.7%)	76 (63.9%)	56 (49.6%)	0.043	0.740	0.033
Intracranial ICA	159 (3.9%)	142 (3.7%)	8 (6.7%)	9 (8%)	0.019	0.179	0.042
MCA M1	329 (8.1%)	305 (8%)	10 (8.4%)	14 (12.4%)	0.232	0.980	0.169
MCA M2-4	621 (15.3%)	586 (15.3%)	12 (10.1%)	23 (20.4%)	0.094	0.227	0.259
ACA A1-2	43 (1.1%)	40 (1%)	2 (1.7%)	1 (0.9%)	0.786	0.753	0.983
PCA P1-2	148 (3.6%)	139 (3.6%)	3 (2.5%)	6 (5.3%)	0.515	0.776	0.572
BA	78 (1.9%)	73 (1.9%)	3 (2.5%)	2 (1.8%)	0.884	0.862	0.993
V4	68 (1.7%)	65 (1.7%)	2 (1.7%)	1 (0.9%)	0.803	1.000	0.758
Other	160 (3.9%)	156 (4.1%)	3 (2.5%)	1 (0.9%)	0.166	0.631	0.165
Tandem lesion	169 (4.6%)	156 (4.5%)	6 (5.6%)	7 (7.1%)	0.413	0.822	0.393
Last-Time-Seen-Well-to-needle time	183.5 (132.7)	183.2 (132.8)	190.3 (134.3)	188.6 (127.1)	0.723	0.768	0.854

Values are presented as median (interquartile range) or as numbers (proportions); mRS, modified Rankin scale; NIHSS, National Institutes of Health Stroke Scale; BP, blood pressure; ASPECTS, Alberta Stroke Program Early CT score; pc-ASPECTS, posterior circulation ASPECT score; ICA, internal carotid artery; MCA M1/2/3/4, first, second, third and fourth segments of middle cerebral artery; ACA1-2, first and second segments of anterior cerebral artery; PCA P1-2, first and second segments of posterior cerebral artery BA, basilar artery; V4, fourth segment of vertebral artery.

Table S3. Baseline stroke characteristics and imaging data for the EVT cohort, the COVID-19 negative, symptomatic, and asymptomatic SARS-CoV-2 infection groups.

Variables	Total (n=9279)	COVID negative (n=8756)	Asymptomatic (n=228)	Symptomatic (n=295)	P-value (Overall group comparison)	P-value (asymptomatic vs. COVID negative)	P-value (symptomatic vs. COVID negative)
Center volume							
< 100	741 (8%)	652 (7.4%)	38 (16.7%)	51 (17.3%)	0.000	0.000	0.000
100-199	2696 (29.1%)	2523 (28.8%)	75 (32.9%)	98 (33.2%)	0.113	0.328	0.192
200-299	3677 (39.6%)	3517 (40.2%)	78 (34.2%)	82 (27.8%)	0.000	0.134	0.000
>=300	2165 (23.3%)	2064 (23.6%)	37 (16.2%)	64 (21.7%)	0.028	0.019	0.701
Demographics					0.000		
Age	71.2 (13.7)	71.3 (13.7)	69.8 (14.7)	68.3 (13.2)	0.000	0.185	0.000
Sex, male	4545 (49%)	4241 (48.5%)	114 (50%)	190 (64.4%)	0.000	0.873	0.000
Pre-stroke independency (mRS ≤2)	8353 (93.3%)	7869 (93.3%)	212 (93.8%)	272 (94.1%)	0.827	0.944	0.827
Vascular risk factors					0.000		
Arterial hypertension	6432 (69.7%)	6085 (69.8%)	155 (68%)	192 (65.1%)	0.184	0.792	0.153
Diabetes mellitus	2277 (24.7%)	2102 (24.1%)	73 (32%)	102 (34.6%)	0.000	0.013	0.000
Dyslipidemia	4225 (45.8%)	4009 (46.1%)	92 (40.4%)	124 (42%)	0.094	0.163	0.307
Current smoking (or stopped < 2 years)	1954 (21.6%)	1860 (21.8%)	40 (17.6%)	54 (18.4%)	0.130	0.244	0.307
Heart failure	1305 (15.1%)	1225 (15%)	32 (14.6%)	48 (17.3%)	0.570	0.978	0.509
Coronary artery disease	1493 (16.5%)	1407 (16.5%)	42 (19.1%)	44 (15.6%)	0.543	0.523	0.901
Active cancer	419 (5.1%)	396 (5.2%)	9 (4.3%)	14 (5.4%)	0.852	0.832	0.984
Treatment at stroke onset					0.000		
Oral anticoagulants	1785 (19.4%)	1665 (19.1%)	53 (23.2%)	67 (22.7%)	0.100	0.228	0.237
Antiplatelets	2345 (25.4%)	2221 (25.5%)	62 (27.2%)	62 (21.2%)	0.199	0.812	0.175
Statins	2903 (33.3%)	2753 (33.5%)	62 (28.4%)	88 (31.5%)	0.236	0.218	0.739
Stroke characteristics					0.000		
Last-Time-Seen-Well-to-door delay (minutes)	213.6 (238.8)	213.9 (238.6)	206.8 (239.8)	209.4 (245.2)	0.874	0.889	0.943
NIHSS admission	16 (10-20)	15 (10-20)	16 (12-21)	17 (12-21)	0.000	0.015	0.000
Vascular territories							
Carotid	8180 (88.8%)	7711 (88.7%)	208 (91.2%)	261 (89.1%)	0.485	0.412	0.975

Variables	Total (n=9279)	COVID negative (n=8756)	Asymptomatic (n=228)	Symptomatic (n=295)	P-value (Overall group comparison)	P-value (asymptomatic vs. COVID negative)	P-value (symptomatic vs. COVID negative)
Vertebrobasilar	816 (8.9%)	779 (9%)	17 (7.5%)	20 (6.8%)	0.338	0.675	0.369
Multiple territories	218 (2.4%)	203 (2.3%)	3 (1.3%)	12 (4.1%)	0.085	0.534	0.100
Admission systolic BP (mmHG)	149.3 (26.1)	149.6 (26.2)	147.7 (25.2)	141.6 (22.9)	0.000	0.483	0.000
Admission blood glucose (mmol/l)	7.6 (2.9)	7.6 (2.9)	7.9 (3.5)	8.6 (3.6)	0.000	0.186	0.000
Stroke etiology							
Large artery atherosclerosis	1830 (19.7%)	1724 (19.7%)	37 (16.2%)	69 (23.4%)	0.118	0.351	0.219
Cardioembolism	4306 (46.4%)	4086 (46.7%)	105 (46%)	115 (39%)	0.034	0.979	0.018
Small vessels disease	30 (0.3%)	29 (0.3%)	0 (0%)	1 (0.3%)	0.684	0.621	1.000
Dissection	211 (2.3%)	201 (2.3%)	4 (1.8%)	6 (2%)	0.830	0.831	0.946
Other determined cause	415 (4.5%)	348 (4%)	31 (13.6%)	36 (12.2%)	0.000	0.000	0.000
Undetermined	2487 (26.8%)	2368 (27%)	51 (22.4%)	68 (23%)	0.097	0.218	0.239
Acute imaging					0.000		
ASPECTS (or pc-ASPECTS)	9 (8-10)	9 (8-10)	9 (8-10)	8 (7-10)	0.000	0.913	0.000
Most proximal arterial occlusion							
None	72 (0.8%)	70 (0.8%)	1 (0.4%)	1 (0.3%)	0.561	0.780	0.607
Intracranial ICA	1880 (20.6%)	1763 (20.5%)	48 (21.1%)	69 (23.7%)	0.401	0.964	0.330
MCA M1	4478 (49.1%)	4223 (49.1%)	117 (51.5%)	138 (47.4%)	0.647	0.712	0.823
MCA M2-4	1701 (18.6%)	1608 (18.7%)	39 (17.2%)	54 (18.6%)	0.847	0.811	0.998
ACA A1-2	51 (0.6%)	49 (0.6%)	1 (0.4%)	1 (0.3%)	0.853	0.959	0.849
PCA P1-2	134 (1.5%)	127 (1.5%)	4 (1.8%)	3 (1%)	0.770	0.924	0.784
BA	578 (6.3%)	554 (6.4%)	12 (5.3%)	12 (4.1%)	0.226	0.732	0.210
V4	112 (1.2%)	107 (1.2%)	2 (0.9%)	3 (1%)	0.846	0.859	0.936
Other	117 (1.3%)	104 (1.2%)	3 (1.3%)	10 (3.4%)	0.004	0.986	0.002
Tandem lesion	1394 (15.3%)	1303 (15.2%)	38 (16.7%)	53 (18.1%)	0.317	0.781	0.298

Values are presented as median (interquartile range) or as numbers (proportions); mRS, modified Rankin scale; NIHSS, National Institutes of Health Stroke Scale; BP, blood pressure; ASPECTS, Alberta Stroke Program Early CT score; pc-ASPECTS, posterior circulation ASPECT score; ICA, internal carotid artery; MCA M1/2/3/4, first, second, third and fourth segments of middle cerebral artery; ACA1-2, first and second segments of anterior cerebral artery; PCA P1-2, first and second segments of posterior cerebral artery BA, basilar artery; V4, fourth segment of vertebral artery.

Table S4. Univariable outcome comparison between COVID-19 negative, symptomatic, and asymptomatic COVID-19 cohorts.

Variables	Total (n=15124)	COVID-19 negative (n=14275)	Asymptomatic COVID-19 (n=395)	Symptomatic COVID-19 (n=454)	P-value (Overall group comparison)	P-value (asymptomatic vs. COVID-19 negative)	P-value (Symptomatic vs. COVID-19 negative)
SICH	784 (5.2%)	712 (5%)	30 (7.6%)	42 (9.4%)	<0.001	0.047	<0.001
SSAH	300 (2.1%)	269 (2%)	16 (4%)	15 (3.4%)	0.002	0.008	0.080
SICH/SSAH	927 (6.4%)	841 (6.1%)	38 (9.6%)	48 (10.7%)	<0.001	0.010	<0.001
24-hour mortality	209 (1.4%)	178 (1.3%)	10 (2.6%)	21 (4.8%)	<0.001	0.073	<0.001
3-month mortality	2761 (20.3%)	2497 (19.5%)	89 (23.2%)	175 (40%)	<0.001	0.142	<0.001
3-month mRS	3 (1-5)	3 (1-5)	3 (1-5)	4 (2-6)	<0.001	0.002	<0.001
3-month mRS 0-2*	7002 (51.7%)	6691 (52.5%)	168 (43.9%)	143 (32.7%)	<0.001	0.002	<0.001
Radiological parenchymal transformation							
No	10976 (74.4%)	10370 (74.6%)	280 (70.9%)	326 (72.8%)	0.180	0.184	0.623
HI	2617 (17.7%)	2467 (17.7%)	78 (19.8%)	72 (16.1%)	0.378	0.516	0.593
PH	1154 (7.8%)	1067 (7.7%)	37 (9.4%)	50 (11.2%)	0.013	0.386	0.014

Values are presented as median (interquartile range) or as numbers (proportions); SICH, Symptomatic intracerebral hemorrhage; SSAH, Symptomatic subarachnoid hemorrhage; mRS, modified Rankin Scale; HI, hemorrhagic infarction; PH, parenchymal hematoma.

Supplementary Figure legends.

Figure S1. Three-month mRS distribution in asymptomatic and symptomatic COVID-19 groups and in controls, in the IVT-only cohort (A) and EVT cohort (B).

Figure S2. Forest plot of intracranial bleeding complications, mortality and disability comparing patients with asymptomatic and symptomatic COVID-19 with COVID-negative controls in the IVT-only cohort (A) and EVT cohort (B).

OR, Odds ratio; CI, Confidence Interval; IVT, Intravenous thrombolysis; EVT, Endovascular treatment; SICH, Symptomatic intracerebral hemorrhage; SSAH, Symptomatic subarachnoid hemorrhage; mRS, modified Rankin Scale.

Figure S1-A

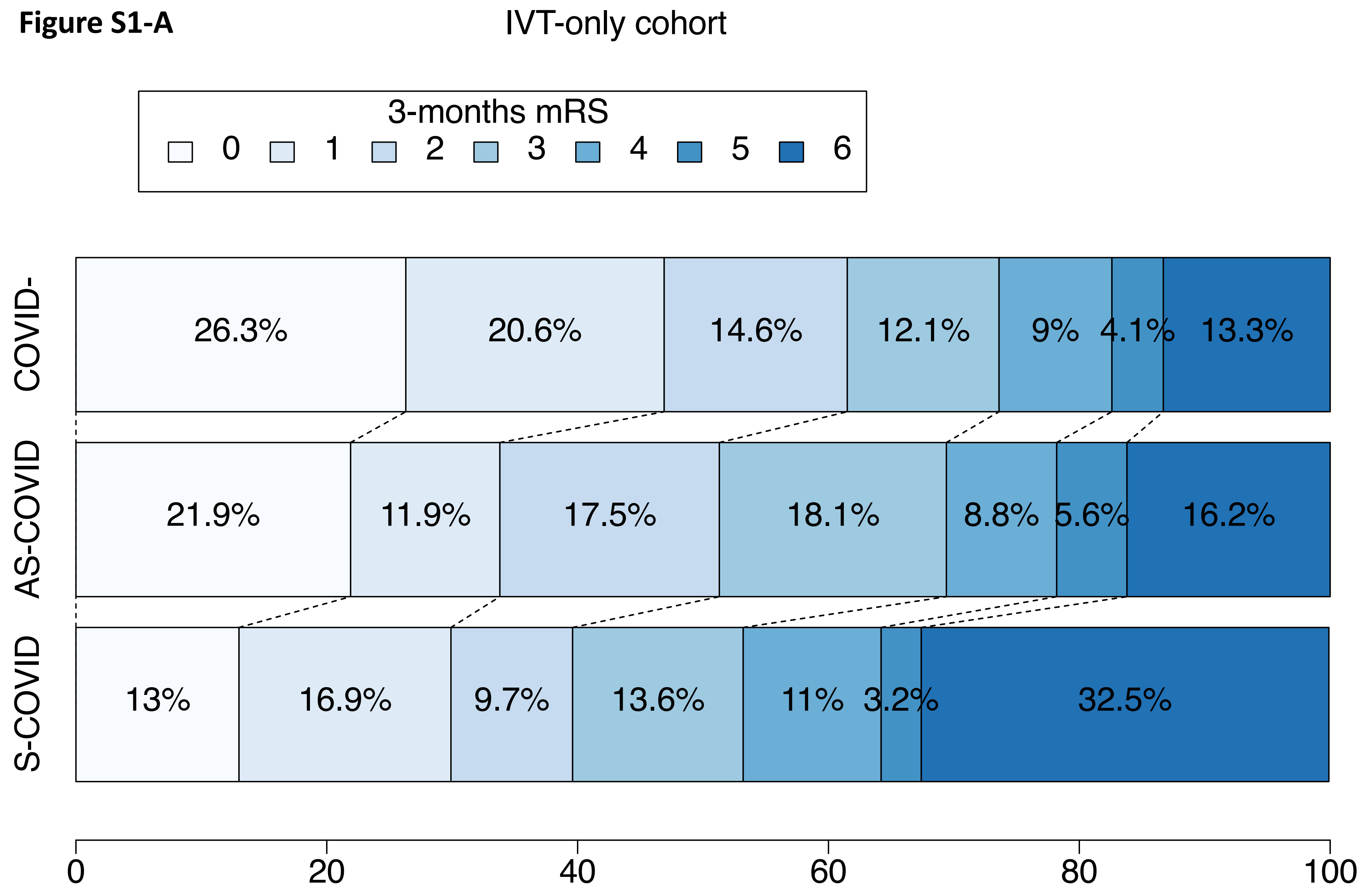
IVT-only cohort
3-months mRS
0
1
2
3
4
5
6
COVID-
26.3%
20.6%
14.6%
12.1%
9%
4.1%
13.3%
AS-COVID
21.9%
11.9%
17.5%
18.1%
8.8%
5.6%
16.2%
S-COVID
13%
16.9%
9.7%
13.6%
11%
3.2%
32.5%
0
20
40
60
80
100

Figure S1-B

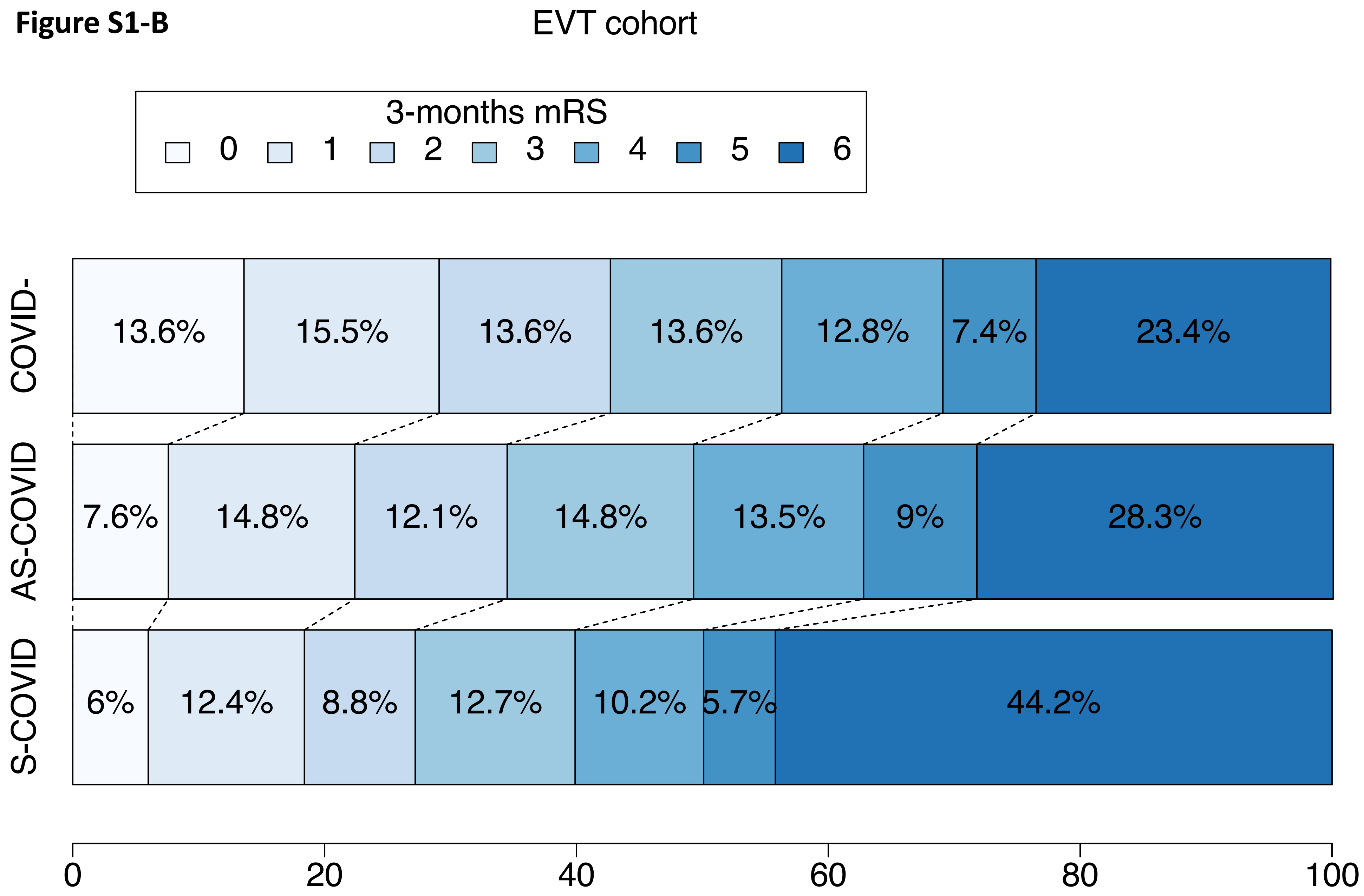

EVT cohort
3-months mRS
0
1
2
3
4
5
6
COVID-
13.6%
15.5%
13.6%
13.6%
12.8%
7.4%
23.4%
AS-COVID
7.6%
14.8%
12.1%
14.8%
13.5%
9%
28.3%
S-COVID
6%
12.4%
8.8%
12.7%
10.2%
5.7%
44.2%
0
20
40
60
80
100

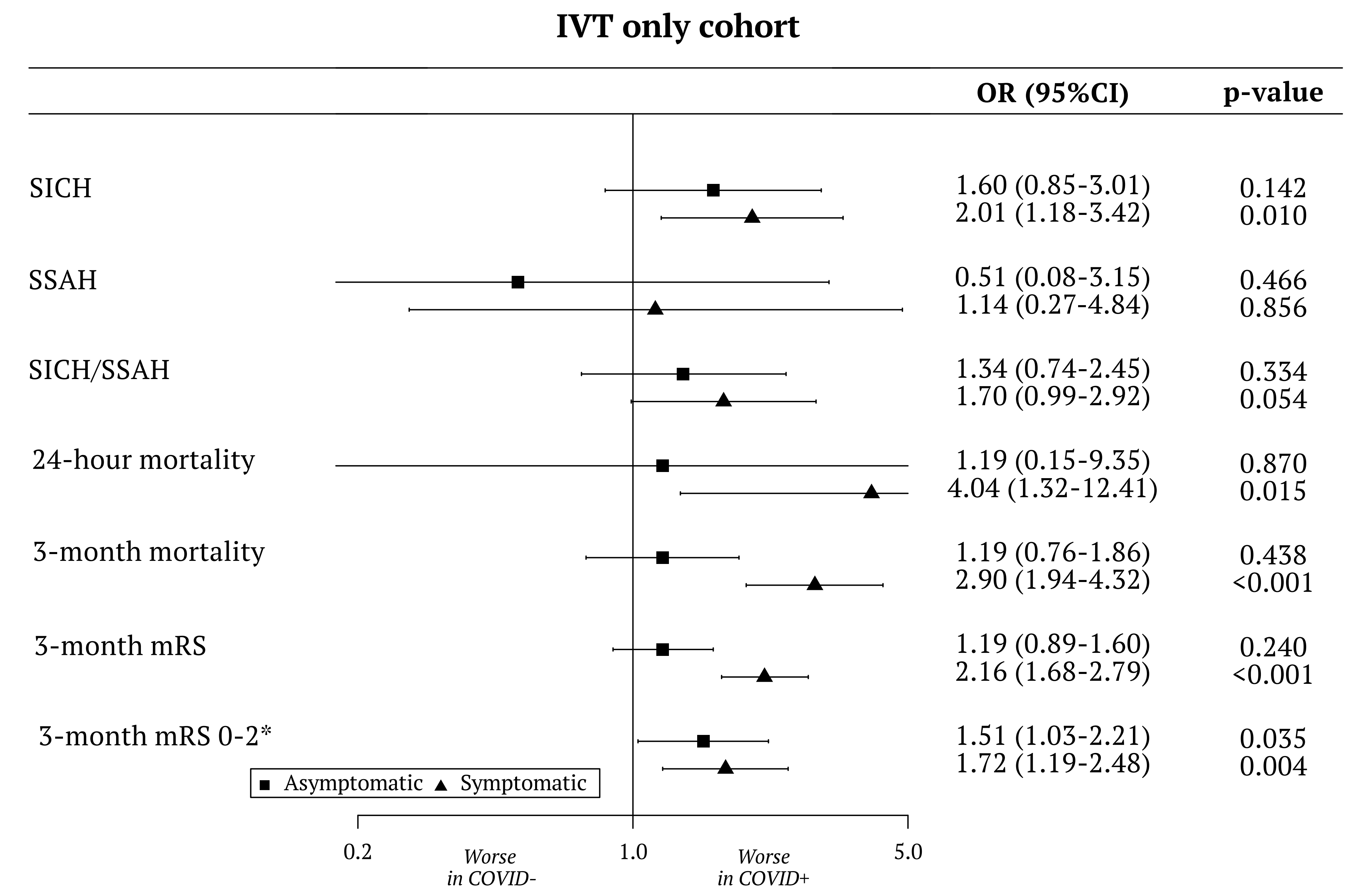
IVT only cohort
OR (95%CI)
p-value
SICH
1.60 (0.85-3.01)
0.142
2.01 (1.18-3.42)
0.010
SSAH
0.51 (0.08-3.15)
0.466
1.14 (0.27-4.84)
0.856
SICH/SSAH
1.34 (0.74-2.45)
0.334
1.70 (0.99-2.92)
0.054
24-hour mortality
1.19 (0.15-9.35)
0.870
4.04 (1.32-12.41)
0.015
3-month mortality
1.19 (0.76-1.86)
0.438
2.90 (1.94-4.32)
<0.001
3-month mRS
1.19 (0.89-1.60)
0.240
2.16 (1.68-2.79)
<0.001
3-month mRS 0-2*
1.51 (1.03-2.21)
0.035
1.72 (1.19-2.48)
0.004
Asymptomatic
Symptomatic
0.2
1.0
5.0
Worse in COVID-
Worse in COVID+

Figure S2-A

EVT cohort

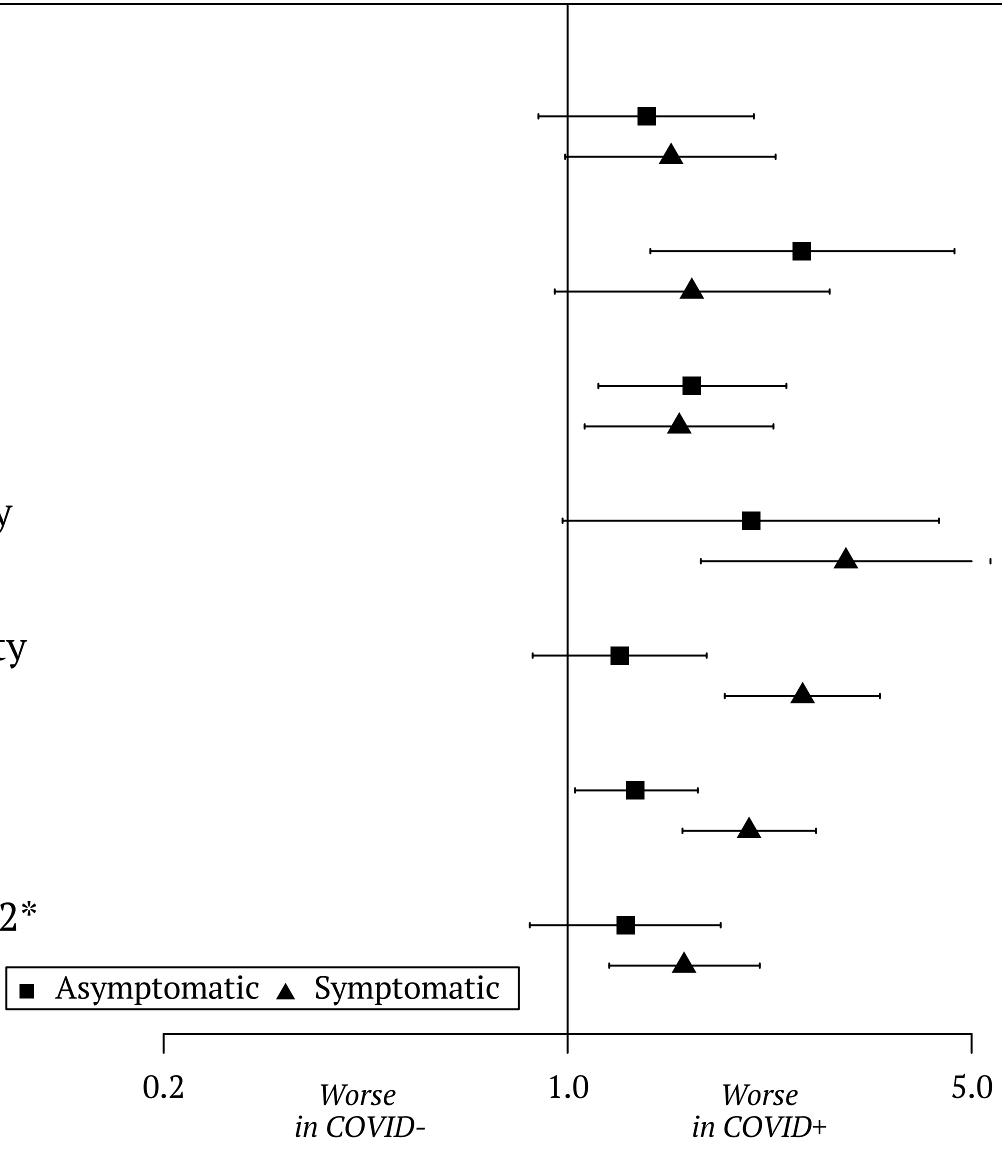

	Group	OR (95%CI)	p-value
SICH	Asymptomatic	1.37 (0.89-2.10)	0.150
	Symptomatic	1.51 (0.99-2.29)	0.053
SSAH	Asymptomatic	2.54 (1.39-4.67)	0.003
	Symptomatic	1.64 (0.95-2.84)	0.076
SICH/SSAH	Asymptomatic	1.64 (1.13-2.39)	0.010
	Symptomatic	1.56 (1.07-2.27)	0.021
24-hour mortality	Asymptomatic	2.08 (0.98-4.39)	0.056
	Symptomatic	3.03 (1.70-5.39)	<0.001
3-month mortality	Asymptomatic	1.23 (0.87-1.74)	0.237
	Symptomatic	2.55 (1.87-3.47)	<0.001
3-month mRS	Asymptomatic	1.31 (1.03-1.68)	0.030
	Symptomatic	2.06 (1.58-2.69)	<0.001
3-month mRS 0-2*	Asymptomatic	1.26 (0.86-1.84)	0.235
	Symptomatic	1.59 (1.18-2.15)	0.003

Figure S2-B
